# The locus coeruleus maintains core body temperature and protects against hypothermia during dexmedetomidine-induced sedation

**DOI:** 10.1073/pnas.2422878122

**Published:** 2025-10-07

**Authors:** Berta Anuncibay Soto, Ying Ma, Mathieu Nollet, Sara Wong, Giulia Miracca, Daniel Rastinejad, Raquel Yustos, Alexei L. Vyssotski, Nicholas P. Franks, William Wisden

**Affiliations:** ^a^Department of Life Sciences, Imperial College London, London SW7 2AZ, United Kingdom; ^b^United Kingdom Dementia Research Institute at Imperial College London, London W12 0BZ, United Kingdom; ^c^Institute of Neuroinformatics, University of Zurich and ETH Zurich, Zurich CH8057, Switzerland

**Keywords:** NREM sleep, sedation, body temperature, locus ceruleus, dexmedetomidine

## Abstract

Activating the *adra2a* receptors of the brain’s norepinephrine (NE) system produces NREM-like sleep and hypothermia. Dexmedetomidine (DEX), a selective α2 receptor (*adra2a*) agonist, is an important drug used in intensive care for arousable sedation. But for some patients, even slight decreases in body temperature during sedation could pose health risks such as increasing the likelihood of postoperative cognitive dysfunction. Where are the *adra2a* receptors at which DEX acts to induce hypothermia? A popular theory is that inhibitory *adra2a* receptors expressed on norepinephrinergic locus coeruleus (LC) neurons are DEX’s prime targets. To examine this, we genetically lesioned *galanin* (*gal*)-expressing norepinephrinergic LC neurons in adult mice. In these *ΔLC* mice, baseline sleep–wake levels were unchanged, but surprisingly, core body temperatures were lower. Maintaining body temperature depended on NE/*gal* projections from the LC to the medial preoptic (MPO) hypothalamus. Furthermore, without these LC neurons, DEX induced hypothermia more strongly. Knockdown with *adra2a shRNA* revealed that DEX induces hypothermia via *adra2a* receptors on MPO glutamate cells. When *ΔLC* mice were kept warm, DEX could induce the same amount of NREM-like sleep as well as it did for mice with an intact LC, and this sleep-like state in *ΔLC* mice had a higher delta frequency power than in control mice. Our results emphasize that inhibition of the LC by DEX is not required to induce robust sedation. By contrast, the LC likely has a fortuitous background action in protecting against drug-induced hypothermia and speeds recovery from hypothermia.

## Significance

Dexmedetomidine (DEX), a widely used sedative in intensive care, induces an arousable state resembling non-rapid eye movement (NREM) sleep and lowers body temperature. For some patients, even slight decreases in body temperature during sedation could pose health risks. It is commonly believed that DEX targets inhibitory adrenergic receptors on norepinephrinergic locus coeruleus (LC) neurons to induce sedation. However, our study in mice challenges this. We found that the LC, via the preoptic hypothalamus, helps maintain body temperature. Without this LC–hypothalamus link, mice are more sensitive to DEX, resulting in deeper hypothermia. The LC is also not needed for the induction of NREM-like sleep by DEX. On the other hand, the LC accelerates recovery from hypothermia and may reduce adverse outcomes related to sedation.

Norepinephrine (NE) is usually associated with arousal, but one branch of the NE system, when selectively activated, produces NREM-like sleep and hypothermia (1). This is exemplified by dexmedetomidine (DEX), an α2 receptor adreno-agonist (2). DEX, an important intensive care drug, produces arousable sedation with a reduced risk of delirium (3–8), and is also widely used in veterinary medicine (9). Electroencephalogram (EEG) measurements show the sleep state induced by DEX in humans resembles stage 2 and 3 NREM sleep, with delta (1 to 4 Hz) waves (10–13). In rodents, DEX similarly produces NREM-like sleep (14–21); and REM sleep is suppressed by DEX in both humans and rodents (10, 18, 19). Further highlighting the similarity between DEX-induced and natural NREM sleep, changes in regional cerebral blood flow evoked by DEX in humans mirror those in natural NREM sleep (22).

In animals and humans, in addition to inducing a NREM-like sleep state, DEX lowers body temperature, and heart and metabolic rates (14–16, 21, 23–25). Indeed, without external warming the state induced by DEX can be viewed as torpor-like (21). During anesthesia and sedation, although every attempt is made to keep patients warm, inadvertent hypothermia (defined clinically as a core body temperature less than 35 °C) is a complication for some patients (26, 27). While hypothermia is beneficial for cardiac surgery and organ transplantation (27), consequences of inadvertent hypothermia can include increased blood loss, increased risk of wound infections and poorer healing, myocardial ischemia, and postoperative cognitive dysfunction (26, 28, 29). The latter may be caused by increased phosphorylation of the microtubule-associated protein tau, which in turn could increase the risk of developing neurodegenerative disease (28, 30). Early after DEX was introduced clinically, it was suggested that the drug disrupts body temperature control, causing an expanded temperature range that does not trigger thermoregulatory responses (31). Thus, it was proposed that patients sedated with DEX might have an increased risk of hypothermia (31). Although hypothermia is not usually a clinical complication of DEX, there have been cases where DEX caused mild hypothermia in patients (32, 33).

Total gene knockouts in mice show that *adra2a* receptors mediate DEX’s sedative and hypothermic actions (23, 34). Where in the brain are these *adra2a* receptors that DEX works on? From analyzing the relative timing of delta oscillations in the neocortex and thalamus, DEX initiates NREM-like sleep subcortically (20). Much of the brain’s NE is produced in widely projecting neurons in the locus coeruleus (LC)/group A6 in the brainstem (35). The dominant theory for how DEX induces a NREM sleep-like state is that it binds to *adra2a* autoreceptors on locus ceruleus (LC) neurons to reduce NE release throughout the brain (3, 36–39). For example, direct infusion of a high concentration (165 mM) of DEX into the rat LC induced behavioral sedation as defined by loss-of-righting reflex (38), and indeed, we have found in acute slice recordings that DEX inhibits spontaneous activity of LC cells via *adra2a* receptors (18).

Direct agonist infusion into the LC does not rule out the drug reaching other brain regions via diffusion through the nearby 4th ventricle, however. In rats with NE transmission removed by toxin depletion of NE stores, DEX could still lower the minimum alveolar concentration of halothane needed for anesthesia, suggesting DEX acts postsynaptically of the LC to induce sedation (40). Indeed, we have found by *c-fos* activity tagging, that neurons in the mouse PO hypothalamic area are sufficient for DEX-induced NREM sleep and hypothermia (18). Furthermore, within the lateral PO (LPO) hypothalamic area, genetically lesioning galanin peptide (*gal*)-expressing neurons reduced DEX’s ability to induce NREM-like sleep and hypothermia (19). But although LPO *gal* neurons and other hypothalamic neurons near the supraoptic nucleus (SON), as well as neurons in the suprachiasmatic nucleus, are part of the circuitry by which DEX acts to induce NREM-like sleep (18, 19, 41, 42), these cells themselves might not express the critical *adra2a* receptors. Instead, the prime movers for initiating DEX’s sleep and hypothermic actions could still be *adra2a* receptors expressed on the LC neurons. To test this hypothesis, we selectively lesioned the LC and examined how mice responded to DEX.

Strikingly, without the LC, mice were more hypothermic at baseline and more sensitive to DEX, showing prolonged NREM-like sleep and deeper hypothermia. These effects depended on LC projections to the medial (M) preoptic (PO) hypothalamus, where *adra2a* receptors are expressed. When *ΔLC* mice were kept warm, DEX induced similar NREM-like sleep to controls, but with higher delta power. We conclude that the LC is not required for DEX-induced NREM-like sleep and hypothermia. Genetic knockdown showed that *adra2a* receptors on MPO hypothalamus glutamate cells contribute to DEX’s ability to induce hypothermia. We suggest that NE released from the LC chronically regulates *adra2a* receptor levels in the PO hypothalamus, the consequence being that an intact LC restrains DEX-induced hypothermia and aids recovery from hypothermia.

## Results

### Genetic Lesioning of LC Cells to Generate *ΔLC* Mice.

**Fig. 1.**
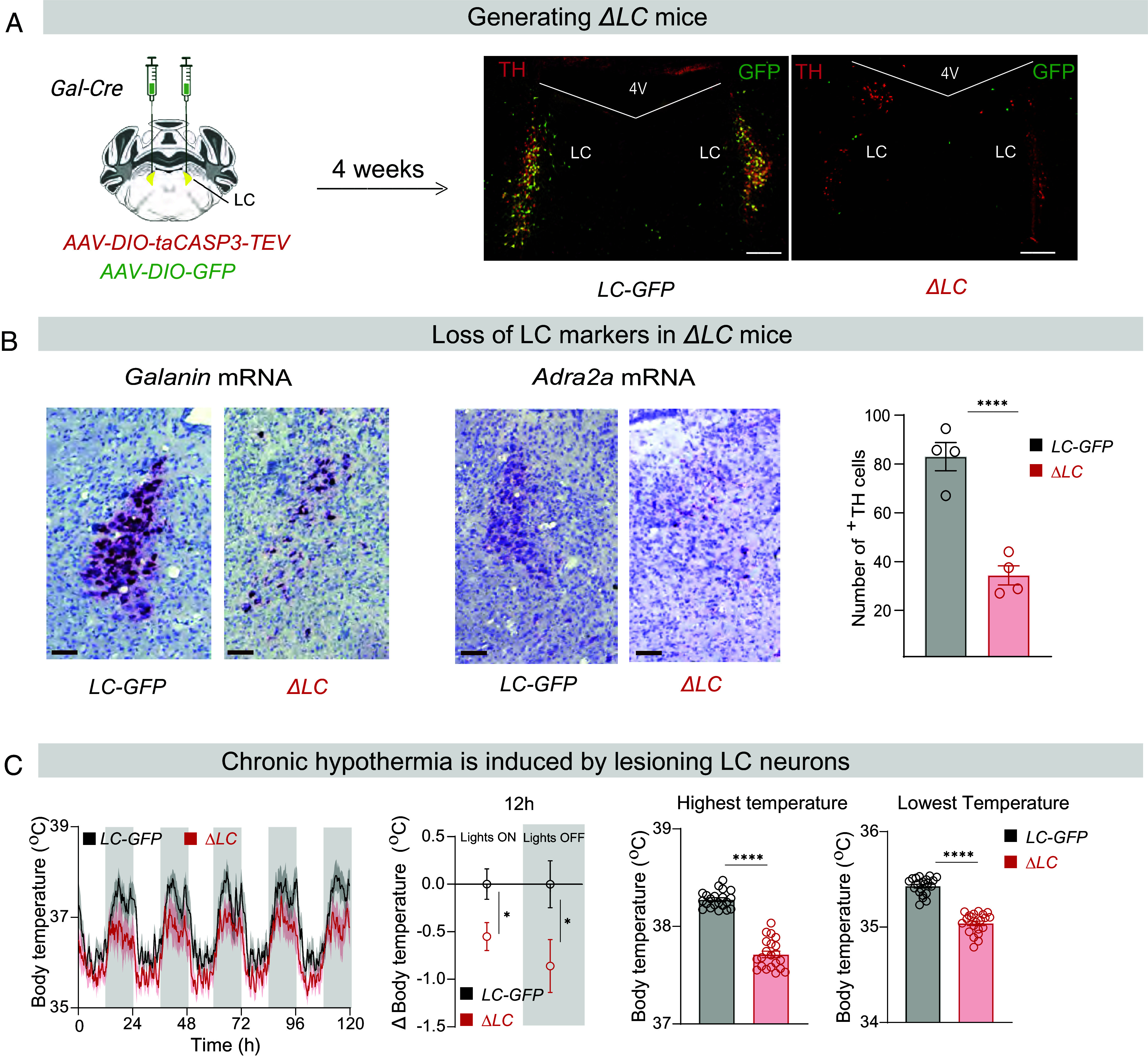
Genetic lesioning of LC neurons produces hypothermia. (*A*) *Top,* Schematic of genetic ablation of LC galanin neurons to produce *ΔLC* mice (*Left*) and demonstration of ablation of TH cells (*Right*). LC, locus ceruleus; 4 V, 4th ventricle. (Scale bar, 1,000 µm.) (*B*) RNAscope images showing loss of *galanin* and *adra2a* mRNAs in the LC region of *ΔLC* mice; bar graph, quantification of ablation of TH cells. Unpaired two-tailed *t* test, ****P < 0.001 (n = 4 in each group). [Scale bar, 100 µm (brown).] (*C*) Lesioning of LC neurons reduces body temperature as assayed over multiple days (n = 7, *ΔLC* and n = 7, *LC-GFP* control mice); middle, average decrease in body temperatures of *ΔLC* mice compared with *LC-GFP* controls (n = 7, unpaired *t* test, *P < 0.05); and *Right*, bar graphs of the highest and lowest temperatures varying over the 24 h cycle, (n = 7 per group, 20 highest or lowest temperature reached over 5 d) Unpaired two-tailed *t* test, ****P < 0.001.

*Galanin* (*gal*) gene expression marks about 80% of norepinephrinergic [tyrosine hydroxylase (TH)-positive] cells in the LC (43–48). Thus, to ablate the LC, we specifically lesioned *Gal*-expressing neurons in the LC of adult *Gal-Cre* mice (Fig. 1*A*). For the experimental group, *AAV-DIO-Caspase* and *AAV-DIO-GFP* viruses were mixed and injected bilaterally into the LC of *Gal-Cre* mice to give *ΔLC* mice (Fig. 1*A*). For controls, *AAV-DIO-GFP* was injected bilaterally into the LC of *Gal-Cre* mice to generate *LC-GFP* mice (Fig. 1*A*). Four weeks after injection, caspase expression had killed approx. 70% of GFP/TH-expressing cells (see bar graph, Fig. 1 *A* and *B* and *SI Appendix*, Fig. S1*A*), substantially reducing *gal* and *adra2a* mRNA content in the LC (Fig. 1*B*). The anatomical characterization of the lesion is shown in *SI Appendix*, Fig. S1*A*.

### Baseline Sleep–Wake States Are Unchanged, but Body Temperature Is Decreased in *ΔLC* Mice.

*ΔLC* mice had the same amounts of wake, NREM and REM sleep as control mice, and there was no change in the frequency of transitions between the three vigilance states (*SI Appendix*, Fig. S1*B*). However, there were significant decreases in the maxima and minima of the diurnal core body temperature profiles of *ΔLC* mice recorded over 5 d compared with the control *LC-GFP* mice (Fig. 1*C*). During the 12 h periods of “lights-off” (mice more active) and “lights-on” (mice less active), *ΔLC* mice were about 1 °C cooler (average core body temperature) in “lights-off” and approx. 0.5 °C cooler during “lights-on” (Fig. 1*C*); *ΔLC* mice had clear decreases in both the highest and lowest body temperatures over the diurnal temperature cycle compared with *LC-GFP* control mice (Fig. 1*C*).

### DEX Potentiates Behavioral Changes in *ΔLC* Mice.

**Fig. 2.**
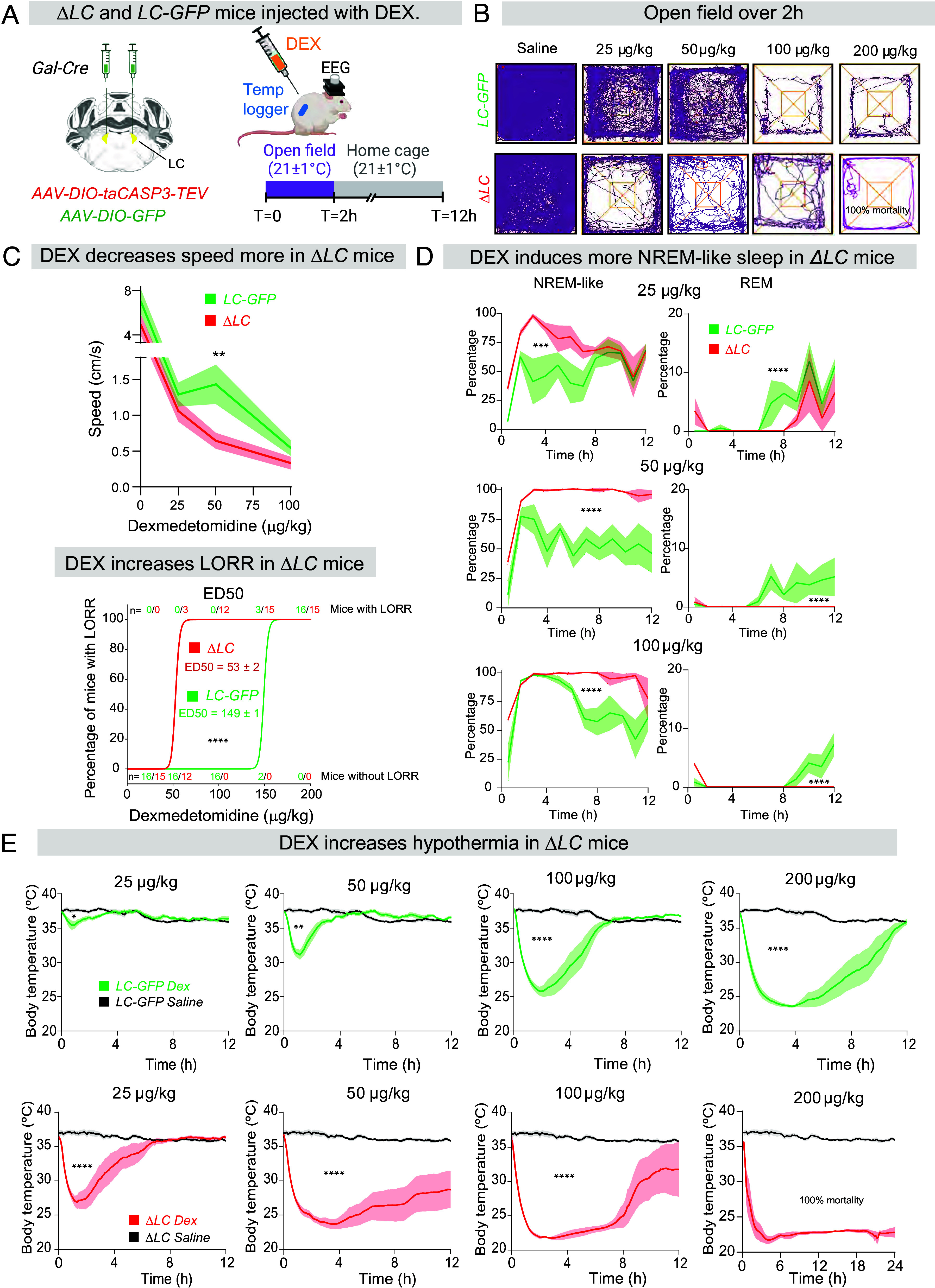
Mice with genetically lesioned LC cells are more sensitive to the sleep and hypothermia-inducing properties of DEX. (*A*) Schematic of genetic ablation of LC neurons to produce *ΔLC* mice, and experimental design. DEX, dexmedetomidine; EEG, electroencephalogram; LC, locus ceruleus. (*B*) Example activity traces recorded over 2 h of individual *ΔLC* and *LC-GFP* mice in the open-field arena with saline and ascending doses of DEX. (*C*) *Upper* graph, speed of *ΔLC* (n = 7) and *LC-GFP* (n = 7) mice in the open-field with ascending doses of DEX. Shading indicates SEM (unpaired one tailed *t* test, **P < 0.01). *Lower* graph, quantal dose–response curve for LORR with DEX for 15 *ΔLC* and 16 *LC-GFP* mice. (****P < 0.001, unpaired two-tailed *t* test). The numbers above and below each data point indicate the number of mice with (*Top* row) and without (*Bottom* row) LORR, respectively, in each group. (*D*) Amounts of wake, NREM-like sleep, and REM sleep over time following saline or ascending doses of DEX injections in *ΔLC* (n = 5) and *LC-GFP* (n = 7) mice (two-way ANOVA, ***P < 0.005; ****P < 0.001). Shading indicates SEM. (*E*) Kinetics of hypothermia induced by ascending doses of DEX in *LC-GFP* (*Top* row, n = 7) and *ΔLC* (*Bottom* row, n = 7) mice compared with unchanged body temperatures after saline injection (Shading indicates SEM; two-way ANOVA, *P < 0.05; **P < 0.01; ***P < 0.005; ****P < 0.001).

We examined how DEX induces behavioral changes in *ΔLC* mice and the *LC-GFP* controls (Fig. 2*A*). We tested a range of DEX concentrations, injected i.p., from 25 µg/kg (amount drug/body weight) through to 200 µg/kg. An immediate behavioral difference between *ΔLC* and *LC-GFP* controls was apparent. In the open-field test, following DEX injection, *ΔLC* mice tracked over 2 h were visibly affected by low doses of DEX (25 µg/kg) compared with *LC-GFP* mice (Fig. 2*B*), moving more slowly (Fig. 2 *C*, *Upper*). (Note: After receiving 200 µg/kg DEX, all the *ΔLC* mice died, whereas *LC-GFP* mice survived). The increased sensitivity to DEX of *ΔLC* mice was also reflected in an approx. threefold left-shift in the quantal dose–response curve for the loss-of-righting reflex (LORR) induced by DEX (Fig. 2 *C*, *Lower* panel; DEX ED_50_ for LORR in *LC-ΔGal* mice, 53 ± 2 µg/kg; and 149 ± 1 µg/kg for *LC-GFP* mice, measured 90 min after DEX injection).

### DEX Induces More NREM-Like Sleep in *ΔLC* Mice.

We next looked at how DEX affects sleep states (Fig. 2*D*). Compared with saline i.p. injections of *ΔLC* and *LC-GFP* mice, which had no effect on sleep–wake states (*SI Appendix*, Fig. S1*C*), DEX i.p. injections produced strong NREM-like sleep, starting 15 min after injection (Fig. 2 *D*, *Left*-hand graphs); however, for inducing and sustaining sleep, DEX was more potent in *ΔLC* mice than in *LC-GFP* mice (Fig. 2*D*); in the first 2 to 3 h postinjection, 25 µg/kg DEX already produced 100% NREM-like sleep in *ΔLC* mice, but for this dose in *LC-GFP* control mice, sleep was partial (see *SI Appendix*, *Materials and Methods*, “*Sleep Scoring*,” for how we classified the mice as being in a “NREM-like” state, particularly when body temperatures were low). As the DEX dose was increased, 50 µg/kg DEX gave 100% NREM-like sleep for at least 8 h in *ΔLC* mice, whereas in control mice, again, sleep was more partial. Control mice only achieved 100% NREM-like sleep with 100 µg/kg DEX, but only for the first 4 h (Fig. 2 *D*, *Bottom Left*-hand graph). This difference in potency for how DEX induces sedation between the *ΔLC* mice and *LC-GFP* mice is also apparent from the perspective that DEX suppresses REM sleep (10). As the DEX is metabolized away, the appearance of REM sleep marks the return of some natural sleep episodes. At 25 µg/kg DEX, *LC-GFP* control mice experienced their first REM episodes some 6 h postinjection, whereas for *ΔLC* mice the first REM episodes only started at 8 h postinjection (Fig. 2 *D*, *Right*-hand graphs). At higher DEX doses, *ΔLC* mice never had any REM sleep within 12 h post–DEX injection, whereas for control mice they always experienced REM sleep some 8 h postinjection (Fig. 2 *D*, *Bottom*-*Right* graph). This further illustrates the increased potency of DEX in *ΔLC* mice to induce and maintain a dominant NREM-like sleep.

### DEX Potentiates Hypothermia in *ΔLC* Mice.

DEX produces hypothermia if animals are not warmed postinjection (18, 19). We looked at how DEX influences body temperature in unwarmed animals at room temperature (21 °C external temp) with, and without, an LC. At 25 µg/kg DEX, control *LC-GFP* mice had a small 2 °C ± 1.5 °C decrease in core body temperature lasting for 2 h post–DEX injection compared with those mice injected with saline (Fig. 2 *E*, *Top* row); however, this dose of DEX in *ΔLC* mice produced a 10 °C ± 2 °C drop in body temperature lasting around 6 h (Fig. 2 *E*, *Bottom* row). As the DEX dose was increased, the extent of hypothermia became more marked in *ΔLC* mice compared with controls, and it took substantially longer for the core body temperature of *ΔLC* animals to increase to baseline levels (Fig. 2*E*). These temperature decreases and recoveries were quantified by measuring the areas (“temperature x time”) formed between the temperature-time curves for saline-injected (baseline) and DEX-injected animals (*SI Appendix*, Fig. S1*D*). For *ΔLC* mice, the amount and persistence of hypothermia induced for any given dose of DEX was much greater than that induced in *LC-GFP* control mice (*SI Appendix*, Fig. S1*D*). At 200 µg/kg DEX, *ΔLC* mice failed to recover from a sustained hypothermia which persisted for 24 h (Fig. 2*E*), and there was 100% mortality for this group at this drug dose.

### External Warming Allows *ΔLC* Mice to Respond to DEX in a Similar Way to Controls, and the LC Remains Dispensable for DEX-Induced NREM-Like Sleep.

**Fig. 3.**
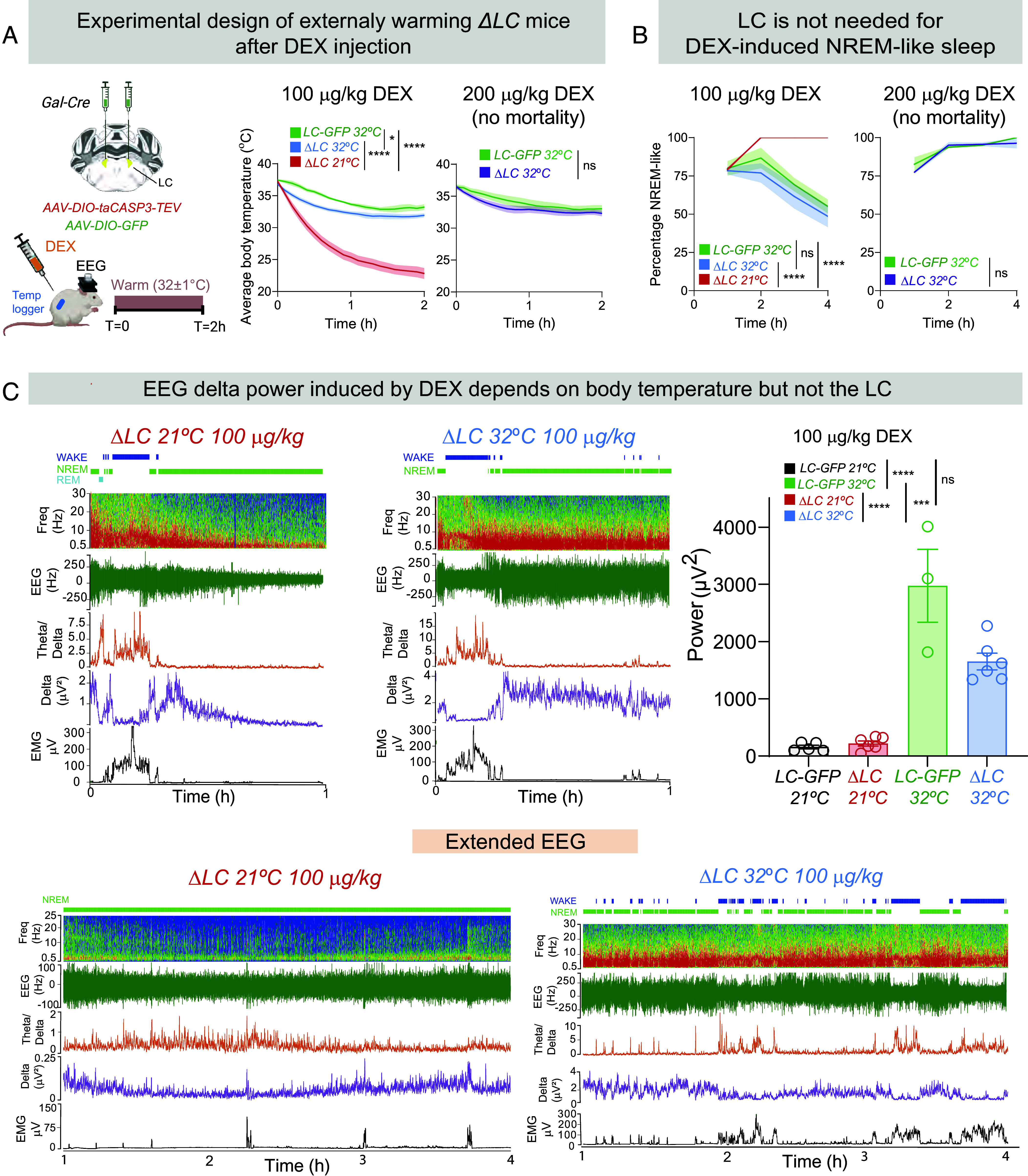
External warming reduces the sensitivity of *ΔLC* mice to DEX, but the LC remains dispensable for DEX-induced sedation. (*A*) Schematic of genetic ablation of LC galanin neurons to produce *ΔLC* mice, and experimental design. EEG, electroencephalogram; LC. locus ceruleus. *Right*-hand graphs: How external warming (32 °C for 2 h after DEX injection) or room temperature (RT 21 °C) influences the time course of DEX-induced hypothermia in *ΔLC* (n = 8 at RT; n = 7 at 32 °C) and *LC-GFP* mice (n = 3 for 32 °C). (two-way ANOVA, *P < 0.05; ****P < 0.001). With external warming after injection, 200 µg/kg DEX is no longer lethal in *ΔLC* mice. (*B*) How external warming (32 °C for 2 h after DEX injection) or room temperature (RT 21 °C) influences the time course of DEX-induced NREM-like sleep in *ΔLC* (n = 8 at RT; n = 6 at 32 °C) and *LC-GFP* mice (n = 3 for 32 °C). (two-way ANOVA, *P < 0.05; ****P < 0.001). (*C*) How body temperature affects the DEX-induced EEG delta power independently of the LC. *ΔLC* (n = 6 at RT; n = 6 at 32 °C) and *LC-GFP* mice (n = 5 for RT and n = 3 for 32 °C). Example traces showing how environmental warming influences the DEX-induced EEG, and in particular increases the EEG delta power in *ΔLC* mice for a given dose of DEX (100 µg/kg) over 1 h post–DEX injection. Note the difference in the delta power scales between the 21 °C and 32 °C examples. Bar graph, quantification of DEX-induced (raw) delta power over the 2-h period post–DEX injection at 21 °C and 32 °C in the *ΔLC* and *LC-GFP* mice (one-way ANOVA, ***P < 0.005; ****P < 0.001). The lower panel, “Extended EEG,” shows the same examples as above, but extended over 4 h.

We tested the effect of DEX in *ΔLC* mice where the external temperature was maintained at 32 °C for the first 2 h following DEX injection (rather than 21 °C used in the previous experiments) (Fig. 3*A*). When DEX injections were done with external warming of *ΔLC* mice at 32 °C, the previously lethal dose of 200 µg/kg DEX was no longer lethal—all *ΔLC* mice survived. Furthermore, with this initial external warming, DEX at 100 µg/kg now only induced a slight (but still significantly) enhanced hypothermia compared with that induced in *LC-GFP* control mice (Fig. 3*A*, compare the green *LC-GFP* and blue *ΔLC* curves; the marked hypothermia induced by the same dose of DEX when mice are at ambient temp 21 °C is shown in red). When *ΔLC* mice with external warming were given 200 µg/kg DEX, their induced hypothermia became further like control *LC-GFP* mice injected with the same DEX dose [Fig. 3*A*, were kept for 2 h in a warm environment postinjection, DEX now induced in *ΔLC* mice a similar amount and time course of NREM-like sleep as seen for control *LC-GFP* mice (Fig. 3), emphasizing that the LC was not needed for DEX’s sedative actions]. On the other hand, with external warming, less NREM-like sleep was induced in *ΔLC* mice experiencing 32 °C for the first 2 h following injection than *ΔLC* mice which were sedated at ambient temperature (Fig. 3 *B*, *Left*-hand trace; and Fig. 2*D*), suggesting that the increased NREM-like sleep in animals maintained only at ambient temperature was a consequence of hypothermia.

### DEX-Induced EEG Delta Power Increases with Body Temperature, but the LC Is Dispensable for DEX-Induced Delta Power.

In *ΔLC* and *LC-GFP* mice injected at ambient temperatures (21 °C) with DEX, the drug induced a NREM-like EEG classification, but after 2 h the EEG had a low power, likely due to the lower temperatures influencing circuit activity (Fig. 3*C*) (see *SI Appendix*, *Materials and Methods*, “*Sleep Scoring*,” for how we classified the mice as being in a “NREM-like” sleep during hypothermia”). Over 4 h post–DEX injection, this low delta power was maintained, but the vigilance state still scored as NREM-like sleep (Fig. 3*C*, “Extended EEG” panel). When the same dose of DEX was given to *ΔLC* mice in a warm environment, however, an approximately ten times larger delta power was produced that also scored as “NREM-like” sleep (Fig. 3 *C*, *Middle* panel and *Right*-hand bar graphs); this high delta power persisted for several hours (Fig. 3*C*, “Extended EEG” panel). Thus, these results emphasize that inhibition of the LC by DEX is not required to induce a robust NREM-like sleep state.

### LC Activity Enhances Emergence from DEX-Induced Sedation and Hypothermia.

**Fig. 4.**
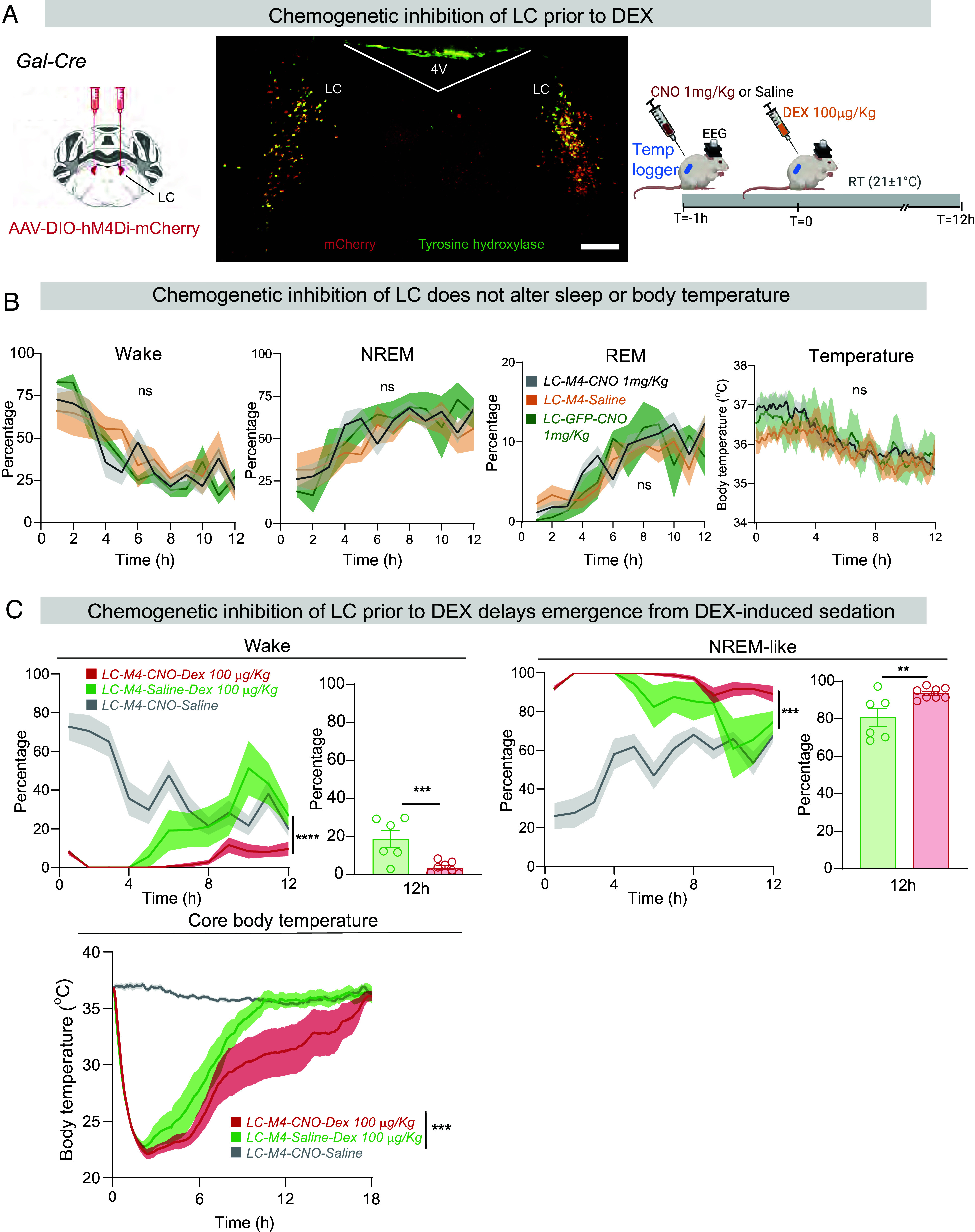
LC activity enhances the emergence from DEX-induced sedation. (*A*) Schematic of CNO-based chemogenetic inhibition of LC cells prior to DEX administration, and immunocytochemical staining for mCherry and TH showing expression of the *hM4Di-mCherry* receptors in TH-positive LC cells in a coronal section. 4 V, 4th ventricle. (Scale bar, 1,000 µm.) (*B*) Chemogenetic inhibition of LC cells with 1 mg/kg CNO but without DEX does not alter baseline sleep–wake or body temperature. Saline + CNO (n = 7) and saline (n = 5) (two-way ANOVA, *P < 0.05). CNO given to *LC-GFP* animals (n = 5), i.e., no chemogenetic receptor present, served as a further control. (*C*) Chemogenetic inhibition of the LC delays the emergence of mice from DEX-induced NREM-like sleep and recovery from hypothermia. CNO+saline (n = 12), CNO+Dex (n = 8), and saline+Dex (n = 6) Curves for wake and sleep, two-way ANOVA, ***P < 0.005, ****P < 0.001, Graphs for wake and sleep, unpaired *t* test one tailed, **P < 0.01, ***P < 0.005; curves for temperature, two-way ANOVA, ***P < 0.005.

We tested whether transient and reversible inhibition of the LC prior to DEX administration influenced how DEX sedated the mice (Fig. 4*A*). The inhibitory DREAD receptor *hM4di* was selectively expressed in the LC by bilaterally injecting *AAV-DIO-hM4Di-mCherry* into the LC of *Gal-Cre* mice, generating *LC-M4* mice (Fig. 4*A*). Compared with *LC-M4* mice given saline injections or *LC-GFP* mice given CNO (1 mg/kg i.p.), chemogenetic inhibition (1 mg/kg CNO i.p.) of the LC had no effect on either sleep or core body temperature (Fig. 4*B*).

To assess the effect of preinhibiting the LC prior to DEX administration, CNO or saline was given 1 h before giving 100 µg/kg DEX (Fig. 4*A*). Induction of NREM-like sleep (at 21 °C external temp) was identical in both groups of mice, as expected if the LC is not needed for DEX to induce NREM-like sleep (Fig. 4*C*); however, mice with chemogenetically inhibited LC cells, plus subsequent DEX administration (Fig. 4*A*), slept 4 h longer than mice given DEX alone (Fig. 4*C*). Following chemogenetic inhibition of the LC, the time course of induction of DEX-induced hypothermia was the same as control mice (Fig. 4*C*), but the recovery of body temperature was slower. Thus, although the LC is not needed for the induction of NREM-like sleep and hypothermia by DEX, it is required for faster emergence of mice from DEX-induced sleep and hypothermia (Fig. 4*C*).

### The LC Maintains Body Temperature by Projections to the Midline and Medial PO Hypothalamus, and These Intact Projections Reduce the Sensitivity of Mice to DEX.

Given that the PO hypothalamus is a key region controlling body temperature (49) and that the LC sends projections to the hypothalamus (50, 51), we hypothesized that the *gal*-expressing LC neurons influence body temperature by their projections to the PO. To test this, we injected *retro-AAV-DIO-caspase*, and *retro-AAV-DIO-GFP (as controls),* into the MPO and MnPO area of *Gal-Cre* mice (*SI Appendix*, Fig. S2*A*). The retro-AAV capsid ensures that the AAV is taken up by LC terminals and transported back to the cell body (52). In the control mice, approx. 0.85% LC neurons expressed GFP, confirming that a subpopulation of *gal*-expressing LC cells send projections to the MPO and MnPO areas (*SI Appendix*, Fig. S2*A*). In the corresponding experimental group, where LC terminals in the MPO/MnPO area had taken up the *retro-AAV-DIO-caspase*, body temperature of the mice was chronically lower. As was the case for the *ΔLC* mice, there were significant decreases in the maxima and minima of the body temperatures of *retro-caspase-LC*-lesioned mice recorded over 5 d compared with the control *retro-caspase*-*LC-GFP* mice, but the decrease in body temperature was confined to the “lights-off” period (*SI Appendix*, Fig. S2*B*). During “lights-off,” *retro-caspase–LC-*lesioned mice were about 1 °C cooler (average core body temperature) (*SI Appendix*, Fig. S2*B*), with decreases in both the highest and lowest body temperatures during “lights-off” compared with *retro*-*LC-GFP* controls (*SI Appendix*, Fig. S2*B*). Baseline sleep–wake profiles were not altered (*SI Appendix*, Fig. S2*C*). However, *retro-caspase–LC-*lesioned animals had a higher sensitivity to DEX (100 µg/kg): Compared with *retro-caspase*-*LC-GFP* controls, *retro-caspase–LC-*lesioned mice stayed in NREM-like sleep longer (probably because of hypothermia), and experienced a slower recovery from hypothermia (*SI Appendix*, Fig. S2*D*). These effects were similar to the increased sensitivity of *ΔLC* mice to DEX (Fig. 2*D*).

### DEX Induces Hypothermia via *adra2a* Receptors Expressed on Glutamate Neurons in the Medial PO Hypothalamus.

The LC has a strong expression of *adra2a* receptors (Fig. 1*B*). But having found the LC and its associated *adra2a* receptors are dispensable for DEX-induced sleep and hypothermia, we looked for relevant DEX targets (i.e., *adra2a* receptors) in the forebrain. There, *adra2a* gene expression occurs mainly in layer VI cortical pyramidal cells, lateral septum (LS) and MPO hypothalamus (*SI Appendix*, Fig. S2*E*) (17, 53). The enhanced sensitivity of LC-lesioned (*ΔLC)* mice to DEX could arise from upregulation of *adra2a* receptors because of a reduced NE input from the LC. Indeed, *adra2a* mRNA levels in *ΔLC* mice were increased about twofold in both medial PO hypothalamus and LS (*SI Appendix*, Fig. S2*F*).

We previously generated and tested a knockdown *adra2a shRNA* that blocked the actions of DEX on LC cells (18). We used the same *adra2a shRNA*, modified into an AAV transgene that was *Cre recombinase*-dependent, to knock down *adra2a* gene expression selectively in glutamate and GABA neurons of the LS and MPO hypothalamic area of *VGlut2-Cre* and *Vgat-Cre* mice (that had nonlesioned LCs). *AAV-DIO-shRNA-adra2a* and *AAV-DIO-shRNA-scramble* (*scr*), each mixed with *AAV-DIO-mCherry* (to mark the sites of shRNA transgene expression), were injected bilaterally into the LS and M (medial) PO hypothalamus (each injection covered both the MPO and septal sites) of *Vgat-Cre* and *Vglut2-Cre* mice to generate *Δadra2a-MPO/septum-Vglut2* and *Δadra2a-MPO/septum-Vgat* mice, respectively. The amounts and distribution of baseline sleep of *Δadra2a-MPO/septum-Vgat* and *Δadra2a-MPO/septum-Vglut2* mice was unchanged from *scr shRNA* controls, and there was no difference in core body temperature compared with controls (*SI Appendix,* Fig. S3 *A* and *B*).

In the *Δadra2a-MPO/septum-Vgat* mice (*SI Appendix*, Fig. S4*A*), *adra2a shRNA* transgene expression was strong in both the LS and MPO areas, reflecting the expression of the endogenous *vgat* gene (*SI Appendix*, Fig. S4*A*). *Δadra2a-MPO/septum-Vgat* and *scr* control mice (ambient temp was 21 °C) were given 25, 50, and 100 µg/kg DEX. At all doses, there was no difference between mouse groups in DEX’s ability to induce NREM-like sleep or hypothermia, and recovery time was also unchanged (*SI Appendix*, Figs. S3*C* and S4*C*). Thus, GABA neurons in the MPO area do not contribute to *adra2a* receptor agonist actions for hypothermia or NREM-like sleep.

**Fig. 5.**
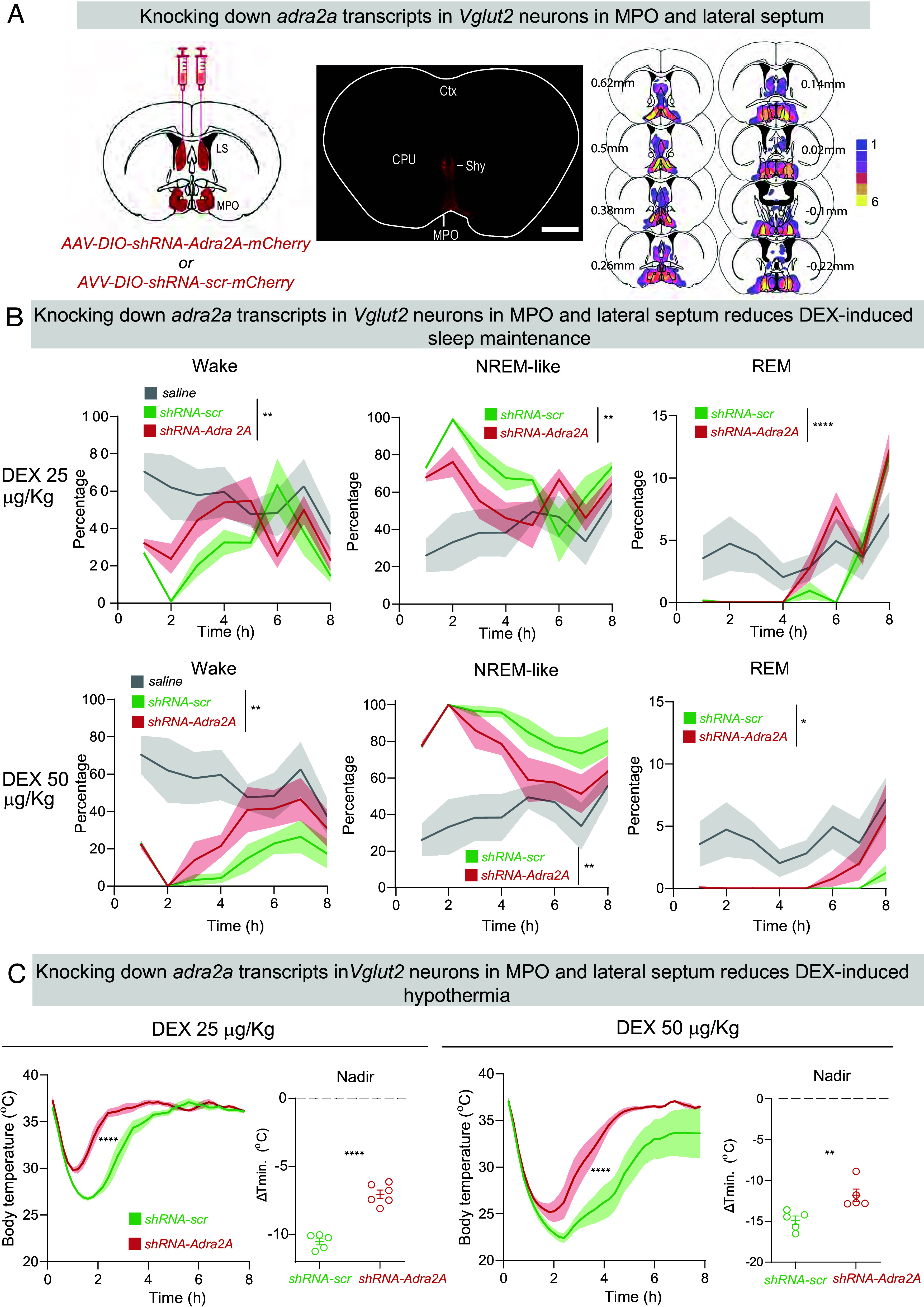
DEX induces hypothermia via *adra2a* receptors expressed on glutamatergic neurons in the MPO hypothalamus. (*A*) *Left*-hand panel: Schematic of knockdown of *adra2a* receptor transcripts in the MPO area of *Vglut-2-Cre* mice. *Middle* panel: expression of *AAV-adra2a-shRNA* transgene in the medial preoptic area (MPO), LS, and septo-hypothalamic (SHy) nucleus, as detected by immunocytochemical staining for mCherry (coronal section). *Right*-hand panel: Heatmap documenting the pooled distribution of *AAV-adra2a-shRNA* transgene expression in n = 6 mice, as detected by mCherry staining. Color key indicates the number of mice that had transgene expression in the area. CPu, Caudate putamen; Ctx, Cortex. (Scale bar, 1,000 µm.) (*B*) DEX-induced NREM-like sleep maintenance is diminished by knocking down *adra2a* receptor expression in MPO glutamatergic cells. Amounts of wake, NREM-like, and REM sleep over time following saline or ascending doses of DEX injections in *Δadra2a-MPO/septum-Vglut-2* (n = 5) and controls (scr) (n = 5) mice (two-way ANOVA, *P < 0.05, **P < 0.01, ****P < 0.001). Shading indicates SEM. Mice were at ambient temperature. (*C*) DEX-induced hypothermia produced with 25 µg/kg and 50 µg/kg DEX requires *adra2a* receptors on MPO glutamatergic neurons. Curves for temperature and time following DEX i.p. injections in *Δadra2a-MPO/septum-Vglut-2* (n = 5) and controls (scr) (n = 5) mice, two-way ANOVA, ****P < 0.001; graphs show the minimum body temperature (nadir) induced by each dose of DEX, which occurred at approx. 2 h after injection (nonpaired *t* test, ****P < 0.001 for 25 mg/kg DEX; and **P < 0.01 for 50 µg/kg DEX). Mice were at ambient temperature.

In *Δadra2a-MPO/septum-Vglut2* mice, *adra2a shRNA* transgene expression was mainly restricted to the MPO hypothalamus and the septal hypothalamic nucleus (there are few endogenous *Vglut2*-expressing cells in the LS, hence little AAV transgene expression there) (Fig. 5*A*). *Scr* and *shRNA* mouse groups were given 25, 50, and 100 µg/kg doses of DEX. At 100 µg/kg, there was no difference between the groups (*SI Appendix*, Fig. S3*C*), but the lower doses of 25 and 50 µg/kg DEX were less effective in maintaining NREM-like sleep in *Δadra2a-MPO/septum-Vglut2* mice, although both doses *induced* sleep equally well as mice treated with *scr shRNA* controls (Fig. 5*B*). There was, with both doses, the same amount of induced NREM-like sleep as found in *scr* mice, but within 30 min *Δadra2a-MPO/septum-Vglut2* mice had started to wake up again compared with *scr* controls (Fig. 5*B*). By 8 h post–DEX injection, the wakefulness of *Δadra2a-MPO/septum-Vglut2* mice injected with DEX were already similar to saline-injected controls. By contrast, the *scr* control mice injected with DEX were still sedated at 8 h postinjection.

DEX at 25 and 50 µg/kg doses was less effective in inducing hypothermia in *Δadra2a-MPO/septum-Vglut2* mice (Fig. 5*C*): The minimum temperature decrease was lower in the knockdown groups e.g., at 25 μg/kg, DEX caused an approx. minus 6 °C drop in body temperature, whereas in the control mice, the drop was around minus 11 °C (Fig. 5*C*). The recovery of body temperature was also faster in the *Δadra2a-MPO/septum-Vglut2* mice compared with *scr* mice (Fig. 5*C*). Thus, a component of how DEX induces hypothermia resides in *adra2a-*expressing *Vglut2* neurons in the MPO/septal hippocampal area.

Is the NREM-like sleep apparently evoked by *adra2* receptors on MPO *Vglut2* cells independent of temperature or a consequence of the hypothermia? To test this, following injection of DEX (25 and 50 µg/kg), *Δadra2a-MPO/septum-Vglut2* and *scr* mice were given 2 h of external warming (*SI Appendix*, Fig. S5*A*). This external warming normalized the difference in sleep time for *Δadra2a-MPO/septum-Vglut2* mice compared with *scr* controls that received the same DEX doses (*SI Appendix*, Fig. S5*B*). Thus, *adra2a* receptors on *vglut2*-expressing MPO neurons predominantly regulate DEX-induced hypothermia rather than NREM-like sleep.

## Discussion

DEX, an important sedative used in intensive care, induces an arousable state in humans resembling (stages 2 and 3) NREM sleep (10–13). A long-standing and popular theory is that DEX induces sedation by inhibiting the LC, a major source of NE (36–39). We unexpectedly find that the LC helps maintain body temperature via NE/gal projections to the MPO hypothalamus. Without the LC, mice with no external warming become hypersensitive to DEX, causing deeper and longer hypothermia. This is likely because the key *adra2a* receptors regulating temperature and NREM sleep are not on the LC but elsewhere in the brain, including in the MPO hypothalamus (19, 54), and become upregulated in the absence of NE released from the LC (*SI Appendix*, Fig. S6). Without the LC, DEX has a greater effect on upregulated *adra2a* receptors to induce NREM-like sleep and hypothermia. When *ΔLC* mice are kept warm, DEX induces the same amount of NREM-like sleep as in mice with an intact LC, but with higher delta power. Our results show LC inhibition by DEX is not required for sedation. However, the LC speeds recovery from hypothermia. Thus, rather than being DEX’s prime target, the LC can be regarded instead as a “brake” on DEX’s hypothermic effects.

In agreement with earlier work where the LC was lesioned in rodents and cats (55, 56), we found LC lesioning does not affect baseline occurrence of sleep–wake states or the amount of sleep–wake fragmentation. Similarly, mice with selective deletion of the vesicular monoamine transporter 2 (*vmat2*) gene from all NE neurons (not just those in the LC), so that the cells cannot release NE, had no changes in the amounts of NREM, REM, and wake (57). We found that chemogenetically inhibiting the LC with CNO acting on hM4di receptors in LC neurons, mimicking DEX’s Gi inhibition, did not induce sleep or hypothermia, suggesting DEX likely does not act at the LC to induce NREM-like sleep. Similarly, opto-inhibiting LC cells does not cause sleep (58). The most distinct sleep-related trait in long-term LC-lesioned animals is their tendency to fall asleep more rapidly in unfamiliar environments (59). Giving *ΔLC* mice DEX, and providing external warming during and following the injections, induced much stronger NREM-like oscillations in the EEG compared with the delta amplitude when *ΔLC* mice at ambient temperatures were given DEX, further emphasizing that the LC is not needed for DEX-induced NREM-like sleep. Similarly, in rats with NE transmission removed by toxin depletion, DEX still lowered the halothane dose (minimum alveolar concentration) needed for anesthesia, suggesting DEX acts postsynaptically of the LC to induce sedation (40).

So, what is the LC doing with regard to DEX? In terms of general sleep–wake control, others have found that the LC aids waking up from stimuli: Optogenetically exciting LC neurons or their terminals in the PO hypothalamus induces waking from sleep (58, 60), and LC activity, by releasing NE into the thalamus during NREM sleep, aids arousability (61); chemogenetically exciting LC cells even induces arousal from general anesthesia (62); conversely, opto-inhibiting LC cells reduces the effectiveness of sounds to wake animals from NREM sleep (63). These observations are all consistent with the LC being required to reduce duration of DEX-induced NREM-like sleep. Chemogenetically inhibiting the LC prior to DEX administration substantially lengthened the duration of DEX-induced hypothermia and NREM-like sleep. As the effect of DEX is wearing off (i.e., DEX is metabolized), the LC is providing an arousal signal. An important part of this arousal effect is likely to be due to promoting recovery of normal body temperature.

Why are *ΔLC* mice more sensitive to DEX? Many types of cells upregulate receptors for a missing neurotransmitter. For example, denervating skeletal muscle elicits large increases in postsynaptic nicotinic acetylcholine receptor expression (64–66). Similarly, in *ΔLC* mice, we speculate that the loss of NE tone causes the upregulation of *adra2a* receptors expressed on forebrain and hypothalamic neurons, making the associated circuits hypersensitive to the remaining NE input, including from other NE groups, such as the A1/C1 or A2/C2 brainstem groups that project to the forebrain (*SI Appendix*, Fig. S6). Because of the upregulated *adra2a* receptors on effector neurons in the LC-lesioned mice, DEX when given to these mice drives down temperature further and produces more hypothermia than in control mice. When mice without an LC are injected with DEX and kept warm for several hours, they have the same amount of NREM-like sleep as *LC-GFP* controls, although delta power is increased in the *ΔLC* mice, but this is less sleep than when the mice are sedated at ambient temperature (Fig. 3*B*). When the mice injected with DEX were unwarmed, the prolonged NREM-like sleep illustrated in Fig. 2*D* is likely caused by hypothermia. For example, perhaps clearance and degradation of the drug are delayed when the mice are colder.

DEX’s increased sedative and behavioral actions in *ΔLC* mice likely explain earlier observations on *dopamine β-hydroxylase* knockout mice (67). These mice, that develop from birth without the capacity to synthesize NE anywhere in the body, are behaviorally hypersensitive to DEX: They have about 67% shorter latencies to induce LORR, and anesthesia lasts up to 545% longer, suggesting an intact NE system is dispensable for DEX’s behavioral actions (67). (Temperature and NREM-like sleep were not measured in that study). From our results, the increased behavioral sensitivity of *dopamine β-hydroxylase* knockouts to DEX is likely due to upregulated *adra2a* expression.

*Adra2a* receptors are often inhibitory (68). In a part of the LPO, the ventral LPO hypothalamus, calcium imaging revealed that although systemically administered high doses of DEX (100 to 150 µg/kg, i.p.) inhibited many neurons, these doses also excited neurons that were active in NREM sleep (about 25% of GABA and around 6% of glutamate neurons) (69). Indeed, systemically injected DEX induces cFOS expression in the LS, bed nucleus stria terminalis (BNST), PO, and supraoptic hypothalamic nucleus (18, 41, 70). As cFOS induction requires neuronal excitation, this induction could be explained as originating by DEX inhibiting GABA interneurons that are, in turn, inhibiting the neurons that respond with cFOS induction (i.e., disinhibition). However, DEX can also excite neurons directly, as under some circumstances *adra2a* receptors couple to Gs proteins (68). DEX also directly depolarizes cells by reducing cAMP concentrations (via Gi) and thereby inhibiting hyperpolarization-activated cyclic nucleotide-gated cation channels which act as a clamp on the membrane potential, a mechanism that occurs in BNST neurons neighboring the PO area (71), and could also occur in the PO area too.

The PO hypothalamus is well known for housing circuitry that regulates sleep–wake states and temperature (49, 72). PO GABA cells can both promote and inhibit wake, depending on type and location (19, 21, 73, 74); similarly, PO glutamate cells can promote wake or sleep too, again depending on type and location (75–78), and can induce hypothermia and torpor (77, 79); there is quite a range of the hypothermia-inducing glutamate cells in the PO, but these may actually converge to a few discrete subtypes of neurons which have common molecular markers (80).

What is a possible circuit mechanism for how DEX induces hypothermia? Chemogenetically activating *gal* cells in the LPO area induces NREM and drives down core body temperature substantially (19, 74), in a phenotype remarkably like the actions of DEX. On the other hand, lesioning *gal* LPO neurons reduces the effectiveness of DEX to induce NREM-like sleep and reduce body temperature (19), and the mice have permanently elevated core temperatures and strong-sleep–wake fragmentation (19), implying *gal* LPO cells are part of the “NREM sleep-on” and “temperature decrease” switches. The *gal* LPO cells are likely GABAergic, but a subset could use glutamate (81). Yet *adra2a* receptors on GABA (*vgat*) cells in MPO are not responsible for DEX-induced sleep and hypothermia. Rather, only knockdown of *adra2a* receptors on MPO glutamate (*Vglut2*) cells blunted hypothermia induction, and did not affect sleep induction, and when the animals were externally warmed, DEX-induced NREM-like sleep was unaffected by *adra2a* knockdown on MPO glutamate cells. Thus, the simplest hypothesis is DEX binds to excitatory *adra2a* receptors on MPO glutamate cells, which then excite a subset of LPO *gal* cells to drive down temperature and induce NREM-like sleep (*SI Appendix*, Fig. S6). Also, in LPO, DEX would activate NREM sleep-inducing *gal* cells, but these might be a different subpopulation from the ones driving hypothermia. It could also be that the actual induction of NREM-like sleep (delta waves in the EEG) by DEX requires the *adra2a* receptors expressed on layer VI pyramidal neurons (*SI Appendix*, Fig. S2*E*), as well as neurons in the supraoptic area (SON). The supraoptic area (SON) in the hypothalamus and neurons in the suprachiasmatic (SCN) nucleus may also contribute to NREM-like sleep induction by DEX (41, 42), though these studies (both using 100 µg/kg DEX) did not document body temperature. SON neurons identified by cFOS trapping regulated anesthetic responses from various agents—not just DEX—and natural NREM sleep induction (41). How, and if, these SON and SCN neurons regulate temperature is unclear.

At the highest DEX dose (200 μg/kg, i.p.) in *ΔLC* mice, DEX caused 100% mortality in unwarmed mice—they did not recover from extended hypothermia; but when kept warm, the same dose was tolerated (Fig. 3 *A* and *B*). The cause of death is unknown, but hypothermia and sustained bradycardia are probable factors, probably due to overstimulation of *adra2a* receptors. DEX clearance from the brain and body will also be slower during hypothermia, causing the drug’s effects to persist. What about off-target effects? At 100 μg/kg DEX, *α2a* knockout mice showed no hypothermic response; at 300 μg/kg (higher than our dose), they had marginal responses; and even 1,000 μg/kg DEX produced only a small hypothermic effect (23). Thus, off-target effects of the maximal DEX dose we used are possible, but less likely.

When DEX is given in the clinic, strenuous efforts are made to keep patients warm, but there is, however, still a risk of inadvertent hypothermia and long-term pathological consequences. Hypothermia is a double-edged sword (27). Controlled and induced hypothermia can protect organs (27). On the other hand, there are many complications of hypothermia (26, 28, 29): For example, a two-degree decrease in core body temperature during a surgical operation triples the risk of wound infections (26), and may trigger postoperative cognitive deficits. These could arise because cooler temperatures during anesthesia slow turnover of phosphorylated forms of the microtubule binding protein tau (the phosphatases work less well), potentially seeding neurodegenerative disease (30). Even in healthy humans, a chronically lower core body temperature is associated with increased phosphorylated tau (82). In this respect, based on the results with *ΔLC* mice, a fully functioning LC likely protects against adverse outcomes of anesthesia. Even when *ΔLC* mice were in a warm environment, although the extra hypothermia following DEX administration was lessened, there was still more than in control mice with an intact LC. In Alzheimer’s disease (AD), LC neurons are some of the first to accumulate hyperphosphorylated tau, and up to 80% of LC neurons die eventually (83), which is about the same amount achieved by the genetic LC lesions in our study. This suggests that, because of premature LC damage in people in the early phases of AD, some years before they manifest cognitive deficits, they would be at slightly more risk during general anesthesia.

There are some caveats to our results: The lack of hypothermia in patients sedated with DEX may be attributed, as shown in our experiments, to the presence of an intact LC, or to hypothermia being dependent on environmental temperature. Alternatively, it may be because the clinical doses of DEX are significantly lower than those used in animal studies. For example, in experimental studies, humans are typically infused with doses of DEX in the range 1 to 6 µg/kg/h (24, 84, 85), and in clinical practice, infusion rates of 0.7 to 1.4 µg/kg/h are recommended (see data in refs. 3 and 85). Nevertheless, human volunteers given 2 µg/kg DEX experienced about a one degree drop in their body temperature (24).

Although hypothermia may not be a significant side effect of DEX in clinical practice, our study provides insight into the mechanism by which the drug, by removing temperature control, lowers the temperature threshold for initiating vasoconstriction and shivering in humans (31). Thus, DEX can be used to reduce shivering when given after anesthesia (31, 33, 86). This property of DEX could have practical clinical applications, such as reducing postoperative shivering after general anesthesia (33), providing sedation for neonates with hypoxic-ischemic encephalopathy undergoing therapeutic hypothermia (87), or for sedating cardiac arrest patients receiving targeted temperature management.

In summary, we have shown that the key textbook mechanism for how α2 adrenergic sedatives are supposed to induce NREM-like sleep and hypothermia by inhibiting the LC is unlikely. The hypothermia actions of DEX are mediated in part by *adra2a* receptors on PO hypothalamic glutamate (*Vglut2*) neurons. On the other hand, the LC does promote recovery from hypothermia and is likely to be protective from any adverse effects of hypothermia.

## Materials and Methods

Full details of transgene construction, AAV production, stereotaxic injection coordinates, drug concentrations, EEG and temperature recording, sleep scoring, chemogenetics, histology and immunostaining, RNAscope (Advanced Cell Diagnostics), and statistics are given in *SI Appendix*. Experiments were in accordance with the United Kingdom Home Office Animal Procedures Act (1986) and were approved by Imperial College’s Animal Welfare and Ethical Review Body. Adult mice of both sexes were used. *Gal-Cre* mice were a gift from N. Heintz (The Rockefeller University, NY) and obtained from the Mutant Mouse Regional Resource Center (stock #031060-UCD) (88); *Vgat-Cre* mice (89) and *Vglut-2 Cre* mice (89) were a gift from B. B. Lowell (Harvard Medical School, Boston) and obtained from the Jackson Laboratory (JAX stock #016963 and #016962, respectively). All AAVs (mixed serotype 1/2 and retrocaspase) were produced in-house as described previously (90). The rAAV2 packaging plasmid was a gift from Alla Karpova (Janelia Research Campus, VA) and David Schaffer (University of California, Berkeley, CA) (Addgene plasmid #81070) (52); *pAAV-DIO-GFP* was a gift from John T. Gray (University of California Davis, CA) (Addgene plasmid 32396); *pAAV-EF1α-DIO-taCASP3-TEV* was a gift from Nirao Shah (Stanford University, CA) (Addgene plasmid 45580) (91); *pAAV-DIO-hM4Di-mCherry* was a gift from Bryan L. Roth (University of North Carolina, NC) (Addgene plasmid 44362) (92). For constructing the *Cre* recombinase-dependent *adra2a* shRNA knockdown and *scr* shRNA transgenes, we used the pPRIME system based on an *mir30* expression cassette (93). EEG recordings were made with Neurologger 2A devices (94). Temperature loggers (DST nano, Star-Oddi) were intra-abdominally implanted as described previously (77).

## Supplementary Material

Appendix 01 (PDF)

## Data Availability

Tif and Excell data have been deposited in Figshare (10.6084/m9.figshare.27609399) (95). The *Cre*-dependent *adra2a* shRNA knockdown and *scr* control AAV plasmid constructs have been deposited at Addgene (Addgene IDs 245063 and 245064, respectively) (96, 97).

## References

[1] N. P. Franks, W. Wisden, The inescapable drive to sleep: Overlapping mechanisms of sleep and sedation. Science **374**, 556–559 (2021). 34709918 10.1126/science.abi8372

[2] H. Scheinin, R. Virtanen, E. MacDonald, R. Lammintausta, M. Scheinin, Medetomidine–A novel alpha 2-adrenoceptor agonist: A review of its pharmacodynamic effects. Prog. Neuropsychopharmacol. Biol. Psychiatry **13**, 635–651 (1989). 2571177 10.1016/0278-5846(89)90051-1

[3] M. A. S. Weerink , Clinical pharmacokinetics and pharmacodynamics of dexmedetomidine. Clin. Pharmacokinet. **56**, 893–913 (2017). 28105598 10.1007/s40262-017-0507-7 PMC5511603

[4] K. T. Ng, C. J. Shubash, J. S. Chong, The effect of dexmedetomidine on delirium and agitation in patients in intensive care: Systematic review and meta-analysis with trial sequential analysis. Anaesthesia **74**, 380–392 (2019). 30367689 10.1111/anae.14472

[5] Y. Skrobik , A randomized, placebo-controlled trial. Am. J. Respir. Crit. Care Med. **197**, 1147–1156 (2018). 29498534 10.1164/rccm.201710-1995OC

[6] X. Yu, N. P. Franks, W. Wisden, Sleep and sedative states induced by targeting the histamine and noradrenergic systems. Front. Neural Circuits **12**, 4 (2018). 29434539 10.3389/fncir.2018.00004 PMC5790777

[7] G. M. Keating, Dexmedetomidine: A review of its use for sedation in the intensive care setting. Drugs **75**, 1119–1130 (2015). 26063213 10.1007/s40265-015-0419-5

[8] L. Sattar , Comparison between Dexmedetomidine and Propofol for sedation on outcomes after cardiac surgery in patients requiring mechanical ventilation: A meta-analysis of randomized-control trials. Cureus **15**, e42212 (2023). 37609090 10.7759/cureus.42212 PMC10441820

[9] C. Franco, F. Evangelista, A. Briganti, Multiple uses of dexmedetomidine in small animals: A mini review. Front. Vet. Sci. **10**, 1135124 (2023). 37342619 10.3389/fvets.2023.1135124 PMC10278766

[10] O. Akeju , Dexmedetomidine promotes biomimetic non-rapid eye movement stage 3 sleep in humans: A pilot study. Clin. Neurophysiol. **129**, 69–78 (2018). 29154132 10.1016/j.clinph.2017.10.005 PMC5743618

[11] S. M. Ramaswamy, M. A. S. Weerink, M. Struys, S. B. Nagaraj, Dexmedetomidine-induced deep sedation mimics non-rapid eye movement stage 3 sleep: Large-scale validation using machine learning. Sleep **44**, zsaa167 (2021). 32860500 10.1093/sleep/zsaa167 PMC7879420

[12] E. Huupponen , Electroencephalogram spindle activity during dexmedetomidine sedation and physiological sleep. Acta Anaesth. Scand. **52**, 289–294 (2008). 18005372 10.1111/j.1399-6576.2007.01537.x

[13] K. P. Mason, E. O’Mahony, D. Zurakowski, M. H. Libenson, Effects of dexmedetomidine sedation on the EEG in children. Pediatr. Anesth. **19**, 1175–1183 (2009). 10.1111/j.1460-9592.2009.03160.x 20017865

[14] C. Bol, M. Danhof, D. R. Stanski, J. W. Mandema, Pharmacokinetic-pharmacodynamic characterization of the cardiovascular, hypnotic, EEG and ventilatory responses to dexmedetomidine in the rat. J. Pharmacol. Exp. Ther. **283**, 1051–1058 (1997). 9399976

[15] W. F. Seidel, M. Maze, W. C. Dement, D. M. Edgar, Alpha-2 adrenergic modulation of sleep: Time-of-day-dependent pharmacodynamic profiles of dexmedetomidine and clonidine in the rat. J. Pharmacol. Exp. Ther. **275**, 263–273 (1995). 7562559

[16] E. MacDonald, M. Scheinin, H. Scheinin, R. Virtanen, Comparison of the behavioral and neurochemical effects of the two optical enantiomers of medetomidine, a selective alpha-2-adrenoceptor agonist. J. Pharmacol. Exp. Ther. **259**, 848–854 (1991). 1682487

[17] C. Gelegen , Staying awake–A genetic region that hinders alpha2 adrenergic receptor agonist-induced sleep. Eur. J. Neurosci. **40**, 2311–2319 (2014). 24674448 10.1111/ejn.12570 PMC4215598

[18] Z. Zhang , Neuronal ensembles sufficient for recovery sleep and the sedative actions of alpha2 adrenergic agonists. Nat. Neurosci. **18**, 553–561 (2015). 25706476 10.1038/nn.3957 PMC4836567

[19] Y. Ma , Galanin neurons unite sleep homeostasis and alpha2-adrenergic sedation. Curr. Biol. **29**, 3315–3322.e13 (2019). 31543455 10.1016/j.cub.2019.07.087 PMC6868514

[20] R. Baker , Altered activity in the central medial thalamus precedes changes in the neocortex during transitions into both sleep and propofol anesthesia. J. Neurosci. **34**, 13326–13335 (2014). 25274812 10.1523/JNEUROSCI.1519-14.2014 PMC4180471

[21] T. Yamagata , The hypothalamic link between arousal and sleep homeostasis in mice. Proc. Natl. Acad. Sci. U.S.A. **118**, e2101580118 (2021). 34903646 10.1073/pnas.2101580118 PMC8713782

[22] A. Scheinin , Foundations of human consciousness: Imaging the twilight zone. J. Neurosci. **41**, 1769–1778 (2021). 33372062 10.1523/JNEUROSCI.0775-20.2020 PMC8115882

[23] J. C. Hunter , Assessment of the role of alpha2-adrenoceptor subtypes in the antinociceptive, sedative and hypothermic action of dexmedetomidine in transgenic mice. Br. J. Pharmacol. **122**, 1339–1344 (1997). 9421280 10.1038/sj.bjp.0701520 PMC1565079

[24] J. P. Belleville, D. S. Ward, B. C. Bloor, M. Maze, Effects of intravenous dexmedetomidine in humans. I. Sedation, ventilation, and metabolic rate. Anesthesiology **77**, 1125–1133 (1992). 1361310 10.1097/00000542-199212000-00013

[25] K. Lewis , Dexmedetomidine vs other sedatives in critically ill mechanically ventilated adults: A systematic review and meta-analysis of randomized trials. Intensive Care Med. **48**, 811–840 (2022). 35648198 10.1007/s00134-022-06712-2

[26] D. I. Sessler, Complications and treatment of mild hypothermia. Anesthesiology **95**, 531–543 (2001). 11506130 10.1097/00000542-200108000-00040

[27] T. Tveita, G. C. Sieck, Physiological impact of hypothermia: The good, the bad, and the ugly. Physiology (Bethesda) **37**, 69–87 (2022). 34632808 10.1152/physiol.00025.2021

[28] M. A. Papon, R. A. Whittington, N. B. El-Khoury, E. Planel, Alzheimer’s disease and anesthesia. Front. Neurosci. **4**, 272 (2011). 21344011 10.3389/fnins.2010.00272 PMC3034231

[29] C. Riley, J. Andrzejowski, Inadvertent perioperative hypothermia. BJA Educ. **18**, 227–233 (2018). 33456837 10.1016/j.bjae.2018.05.003 PMC7807998

[30] E. Planel , Anesthesia leads to tau hyperphosphorylation through inhibition of phosphatase activity by hypothermia. J. Neurosci. **27**, 3090–3097 (2007). 17376970 10.1523/JNEUROSCI.4854-06.2007 PMC6672474

[31] P. Talke , Dexmedetomidine does not alter the sweating threshold, but comparably and linearly decreases the vasoconstriction and shivering thresholds. Anesthesiology **87**, 835–841 (1997). 9357885 10.1097/00000542-199710000-00017

[32] S. H. Kim, Y. Sul, J. B. Ye, J. Y. Lee, J. S. Lee, Dexmedetomidine-associated hypothermia in critical trauma: A case report and literature analysis. Medicine **104**, e41349 (2025). 39833034 10.1097/MD.0000000000041349 PMC11749715

[33] S. R. Lewis, A. Nicholson, A. F. Smith, P. Alderson, Alpha-2 adrenergic agonists for the prevention of shivering following general anaesthesia. Cochrane Database Syst. Rev. **2015**, CD011107 (2015). 26256531 10.1002/14651858.CD011107.pub2 PMC9221859

[34] P. P. Lakhlani , Substitution of a mutant alpha2a-adrenergic receptor via “hit and run” gene targeting reveals the role of this subtype in sedative, analgesic, and anesthetic-sparing responses in vivo. Proc. Natl. Acad. Sci. U.S.A. **94**, 9950–9955 (1997). 9275232 10.1073/pnas.94.18.9950 PMC23306

[35] G. R. Poe , Locus coeruleus: A new look at the blue spot. Nat. Rev. Neurosci. **21**, 644–659 (2020). 32943779 10.1038/s41583-020-0360-9 PMC8991985

[36] A. F. T. Arnsten, Y. Ishizawa, Z. Xie, Scientific rationale for the use of alpha2A-adrenoceptor agonists in treating neuroinflammatory cognitive disorders. Mol. Psychiatry **28**, 4540–4552 (2023). 37029295 10.1038/s41380-023-02057-4 PMC10080530

[37] E. N. Brown, K. J. Pavone, M. Naranjo, Multimodal general anesthesia: Theory and practice. Anesth. Analg. **127**, 1246–1258 (2018). 30252709 10.1213/ANE.0000000000003668 PMC6203428

[38] C. Correa-Sales, B. C. Rabin, M. Maze, A hypnotic response to dexmedetomidine, an alpha 2 agonist, is mediated in the locus coeruleus in rats. Anesthesiology **76**, 948–952 (1992). 1350889 10.1097/00000542-199206000-00013

[39] N. D. A. Persson, P. Uusalo, M. Nedergaard, T. J. Lohela, T. O. Lilius, Could dexmedetomidine be repurposed as a glymphatic enhancer? Trends Pharmacol. Sci. **43**, 1030–1040 (2022). 36280451 10.1016/j.tips.2022.09.007

[40] I. S. Segal, R. G. Vickery, J. K. Walton, V. A. Doze, M. Maze, Dexmedetomidine diminishes halothane anesthetic requirements in rats through a postsynaptic alpha-2 adrenergic-receptor. Anesthesiology **69**, 818–823 (1988). 2848424 10.1097/00000542-198812000-00004

[41] L. F. Jiang-Xie , A common neuroendocrine substrate for diverse general anesthetics and sleep. Neuron **102**, 1053–1065.e54 (2019). 31006556 10.1016/j.neuron.2019.03.033 PMC6554048

[42] Y. Zhang , Dexmedetomidine accelerates photoentrainment and affects sleep structure through the activation of SCN(VIP) neurons. Commun. Biol. **7**, 1707 (2024). 39730868 10.1038/s42003-024-07430-9 PMC11680882

[43] A. L. Gundlach, W. Wisden, B. J. Morris, S. P. Hunt, Localization of preprogalanin mRNA in rat brain: In situ hybridization study with a synthetic oligonucleotide probe. Neurosci. Lett. **114**, 241–247 (1990). 2402333 10.1016/0304-3940(90)90570-y

[44] V. R. Holets, T. Hokfelt, A. Rokaeus, L. Terenius, M. Goldstein, Locus coeruleus neurons in the rat containing neuropeptide Y, tyrosine hydroxylase or galanin and their efferent projections to the spinal cord, cerebral cortex and hypothalamus. Neuroscience **24**, 893–906 (1988). 2454419 10.1016/0306-4522(88)90076-0

[45] T. Melander , Coexistence of galanin-like immunoreactivity with catecholamines, 5-hydroxytryptamine, GABA and neuropeptides in the rat CNS. J. Neurosci. **6**, 3640–3654 (1986). 2432203 10.1523/JNEUROSCI.06-12-03640.1986 PMC6568661

[46] S. E. Perez, D. Wynick, R. A. Steiner, E. J. Mufson, Distribution of galaninergic immunoreactivity in the brain of the mouse. J. Comp. Neurol. **434**, 158–185 (2001). 11331523 10.1002/cne.1171

[47] R. P. Tillage , Co-released norepinephrine and galanin act on different timescales to promote stress-induced anxiety-like behavior. Neuropsychopharmacology **46**, 1535–1543 (2021). 33911187 10.1038/s41386-021-01011-8 PMC8208976

[48] B. Mulvey , Molecular and functional sex differences of noradrenergic neurons in the mouse locus coeruleus. Cell Rep. **23**, 2225–2235 (2018). 29791834 10.1016/j.celrep.2018.04.054 PMC6070358

[49] N. L. S. Machado, C. B. Saper, Genetic identification of preoptic neurons that regulate body temperature in mice. Temperature (Austin) **9**, 14–22 (2022). 35655663 10.1080/23328940.2021.1993734 PMC9154766

[50] F. S. Giorgi, A. Galgani, S. Puglisi-Allegra, C. L. Busceti, F. Fornai, The connections of Locus Coeruleus with hypothalamus: Potential involvement in Alzheimer’s disease. J. Neural. Transm. (Vienna) **128**, 589–613 (2021). 33942174 10.1007/s00702-021-02338-8 PMC8105225

[51] L. A. Schwarz , Viral-genetic tracing of the input-output organization of a central noradrenaline circuit. Nature **524**, 88–92 (2015). 26131933 10.1038/nature14600 PMC4587569

[52] D. G. Tervo , A designer AAV variant permits efficient retrograde access to projection neurons. Neuron **92**, 372–382 (2016). 27720486 10.1016/j.neuron.2016.09.021 PMC5872824

[53] M. Scheinin , Distribution of alpha 2-adrenergic receptor subtype gene expression in rat brain. Brain Res. Mol. Brain Res. **21**, 133–149 (1994). 8164514 10.1016/0169-328x(94)90386-7

[54] M. N. Alam, B. N. Mallick, Role of lateral preoptic area alpha-1 and alpha-2 adrenoceptors in sleep-wakefulness and body temperature regulation. Brain Res. Bull. **35**, 171–177 (1994). 7953774 10.1016/0361-9230(94)90099-x

[55] C. Blanco-Centurion, D. Gerashchenko, P. J. Shiromani, Effects of saporin-induced lesions of three arousal populations on daily levels of sleep and wake. J. Neurosci. **27**, 14041–14048 (2007). 18094243 10.1523/JNEUROSCI.3217-07.2007 PMC2975593

[56] B. E. Jones, S. T. Harper, A. E. Halaris, Effects of locus coeruleus lesions upon cerebral monoamine content, sleep-wakefulness states and the response to amphetamine in the cat. Brain Res. **124**, 473–496 (1977). 192414 10.1016/0006-8993(77)90948-9

[57] E. Isingrini , Behavioral and transcriptomic changes following brain-specific loss of noradrenergic transmission. Biomolecules **13**, 511 (2023). 36979445 10.3390/biom13030511 PMC10046559

[58] M. E. Carter , Tuning arousal with optogenetic modulation of locus coeruleus neurons. Nat. Neurosci. **13**, 1526–1533 (2010). 21037585 10.1038/nn.2682 PMC3174240

[59] H. S. Gompf , Locus ceruleus and anterior cingulate cortex sustain wakefulness in a novel environment. J. Neurosci. **30**, 14543–14551 (2010). 20980612 10.1523/JNEUROSCI.3037-10.2010 PMC2989851

[60] H. Antila , A noradrenergic-hypothalamic neural substrate for stress-induced sleep disturbances. Proc. Natl. Acad. Sci. U.S.A. **119**, e2123528119 (2022). 36331996 10.1073/pnas.2123528119 PMC9659376

[61] A. Osorio-Forero , Noradrenergic circuit control of non-REM sleep substates. Curr. Biol. **31**, 5009–5023.e7 (2021). 34648731 10.1016/j.cub.2021.09.041

[62] E. M. Vazey, G. Aston-Jones, Designer receptor manipulations reveal a role of the locus coeruleus noradrenergic system in isoflurane general anesthesia. Proc. Natl. Acad. Sci. U.S.A. **111**, 3859–3864 (2014). 24567395 10.1073/pnas.1310025111 PMC3956184

[63] H. Hayat , Locus coeruleus norepinephrine activity mediates sensory-evoked awakenings from sleep. Sci. Adv. **6**, eaaz4232 (2020). 32285002 10.1126/sciadv.aaz4232 PMC7141817

[64] S. J. Moss, D. M. Beeson, J. F. Jackson, M. G. Darlison, E. A. Barnard, Differential expression of nicotinic acetylcholine receptor genes in innervated and denervated chicken muscle. EMBO J. **6**, 3917–3921 (1987). 3443094 10.1002/j.1460-2075.1987.tb02732.x PMC553869

[65] R. Miledi, The acetylcholine sensitivity of frog muscle fibres after complete or partial devervation. J. Physiol. **151**, 1–23 (1960). 14422356 PMC1363214

[66] J. P. Merlie, K. E. Isenberg, S. D. Russell, J. R. Sanes, Denervation supersensitivity in skeletal muscle: Analysis with a cloned cDNA probe. J. Cell Biol. **99**, 332–335 (1984). 6547444 10.1083/jcb.99.1.332 PMC2275635

[67] F. Y. Hu , Hypnotic hypersensitivity to volatile anesthetics and dexmedetomidine in dopamine beta-hydroxylase knockout mice. Anesthesiology **117**, 1006–1017 (2012). 23042227 10.1097/ALN.0b013e3182700ab9 PMC3628827

[68] R. G. W. Proudman, J. Akinaga, J. G. Baker, The signaling and selectivity of α-adrenoceptor agonists for the human α2A, α2B and α2C-adrenoceptors and comparison with human α1 and β-adrenoceptors. Pharmacol. Res. Perspect. **10**, e01003 (2022). 36101495 10.1002/prp2.1003 PMC9471048

[69] M. Luo , Divergent neural activity in the VLPO during anesthesia and sleep. Adv. Sci. (Weinh) **10**, e2203395 (2023). 36461756 10.1002/advs.202203395 PMC9839870

[70] L. E. Nelson , The alpha2-adrenoceptor agonist dexmedetomidine converges on an endogenous sleep-promoting pathway to exert its sedative effects. Anesthesiology **98**, 428–436 (2003). 12552203 10.1097/00000542-200302000-00024

[71] N. A. Harris , Dorsal BNST α -adrenergic receptors produce HCN-dependent excitatory actions that initiate anxiogenic behaviors. J. Neurosci. **38**, 8922–8942 (2018). 30150361 10.1523/JNEUROSCI.0963-18.2018 PMC6191524

[72] A. R. Adamantidis, L. de Lecea, Sleep and the hypothalamus. Science **382**, 405–412 (2023). 37883555 10.1126/science.adh8285

[73] S. Chung , Identification of preoptic sleep neurons using retrograde labelling and gene profiling. Nature **545**, 477–481 (2017). 28514446 10.1038/nature22350 PMC5554302

[74] D. Kroeger , Galanin neurons in the ventrolateral preoptic area promote sleep and heat loss in mice. Nat. Commun. **9**, 4129 (2018). 30297727 10.1038/s41467-018-06590-7 PMC6175893

[75] A. Mondino , Glutamatergic neurons in the preoptic hypothalamus promote wakefulness, destabilize NREM sleep, suppress REM sleep, and regulate cortical dynamics. J. Neurosci. **41**, 3462–3478 (2021). 33664133 10.1523/JNEUROSCI.2718-20.2021 PMC8051693

[76] J. Smith , Regulation of stress-induced sleep fragmentation by preoptic glutamatergic neurons. Curr. Biol. **34**, 12–23.e5 (2024). 38096820 10.1016/j.cub.2023.11.035 PMC10872481

[77] E. C. Harding , A neuronal hub binding sleep initiation and body cooling in response to a warm external stimulus. Curr. Biol. **28**, 2263–2273.e64 (2018). 30017485 10.1016/j.cub.2018.05.054 PMC6078908

[78] N. L. S. Machado, W. D. Todd, S. Kaur, C. B. Saper, Median preoptic GABA and glutamate neurons exert differential control over sleep behavior. Curr. Biol. **32**, 2011–2021.e13 (2022). 35385692 10.1016/j.cub.2022.03.039 PMC9090993

[79] S. Hrvatin , Neurons that regulate mouse torpor. Nature **583**, 115–121 (2020). 32528180 10.1038/s41586-020-2387-5 PMC7449701

[80] B. A. Upton, S. P. D’Souza, R. A. Lang, QPLOT neurons-converging on a thermoregulatory preoptic neuronal population. Front. Neurosci. **15**, 665762 (2021). 34017237 10.3389/fnins.2021.665762 PMC8130930

[81] J. R. Moffitt , Molecular, spatial, and functional single-cell profiling of the hypothalamic preoptic region. Science **362**, eaau5324 (2018). 30385464 10.1126/science.aau5324 PMC6482113

[82] E. M. Blessing , Association between lower body temperature and increased tau pathology in cognitively normal older adults. Neurobiol. Dis. **171**, 105748 (2022). 35550158 10.1016/j.nbd.2022.105748 PMC9751849

[83] P. Theofilas , Locus coeruleus volume and cell population changes during Alzheimer’s disease progression: A stereological study in human postmortem brains with potential implication for early-stage biomarker discovery. Alzheimer’s Dement. **13**, 236–246 (2017). 27513978 10.1016/j.jalz.2016.06.2362 PMC5298942

[84] M. Anttila, J. Penttilä, A. Helminen, L. Vuorilehto, H. Scheinin, Bioavailability of dexmedetomidine after extravascular doses in healthy subjects. Br. J. Clin. Pharmacol. **56**, 691–693 (2003). 14616431 10.1046/j.1365-2125.2003.01944.x PMC1884292

[85] M. A. S. Weerink , Pharmacodynamic interaction of remifentanil and dexmedetomidine on depth of sedation and tolerance of laryngoscopy. Anesthesiology **131**, 1004–1017 (2019). 31425170 10.1097/ALN.0000000000002882

[86] J. M. Botros , Comparative study between dexmedetomidine and ondansteron for prevention of post spinal shivering. A randomized controlled trial. BMC Anesthesiol. **18**, 179 (2018). 30501612 10.1186/s12871-018-0640-3 PMC6267838

[87] M. Joshi, J. Muneer, L. Mbuagbaw, I. Goswami, Analgesia and sedation strategies in neonates undergoing whole-body therapeutic hypothermia: A scoping review. PLoS One **18**, e0291170 (2023). 38060481 10.1371/journal.pone.0291170 PMC10703341

[88] E. F. Schmidt, L. Kus, S. Gong, N. Heintz, BAC transgenic mice and the GENSAT database of engineered mouse strains. Cold Spring Harb. Protoc. **2013**, pdb.top073692 (2013). 23457350 10.1101/pdb.top073692

[89] L. Vong , Leptin action on GABAergic neurons prevents obesity and reduces inhibitory tone to POMC neurons. Neuron **71**, 142–154 (2011). 21745644 10.1016/j.neuron.2011.05.028 PMC3134797

[90] M. Klugmann , AAV-mediated hippocampal expression of short and long Homer 1 proteins differentially affect cognition and seizure activity in adult rats. Mol. Cell. Neurosci. **28**, 347–360 (2005). 15691715 10.1016/j.mcn.2004.10.002

[91] C. F. Yang , Sexually dimorphic neurons in the ventromedial hypothalamus govern mating in both sexes and aggression in males. Cell **153**, 896–909 (2013). 23663785 10.1016/j.cell.2013.04.017 PMC3767768

[92] M. J. Krashes , Rapid, reversible activation of AgRP neurons drives feeding behavior in mice. J. Clin. Invest. **121**, 1424–1428 (2011). 21364278 10.1172/JCI46229 PMC3069789

[93] F. Stegmeier, G. Hu, R. J. Rickles, G. J. Hannon, S. J. Elledge, A lentiviral microRNA-based system for single-copy polymerase II-regulated RNA interference in mammalian cells. Proc. Natl. Acad. Sci. U.S.A. **102**, 13212–13217 (2005). 16141338 10.1073/pnas.0506306102 PMC1196357

[94] V. N. Anisimov , Reconstruction of vocal interactions in a group of small songbirds. xNat. Methods **11**, 1135–1137 (2014). 10.1038/nmeth.3114 25262206

[95] B. Anuncibay-Soto , The locus coeruleus maintains core body temperature and protects against hypothermia during dexmedetomidine-induced sedation. Figshare. 10.6084/m9.figshare.27609399. Deposited 4 November 2024. PMC12541345 41055995

[96] B. Anuncibay-Soto , pAAV-hsynapsin-DIO-adra2a-shRNA-mCherry. AddGene. https://www.addgene.org/245063/. Deposited 12 August 2025.

[97] B. Anuncibay-Soto *et al*., pAAV-hsynapsin-DIO-adra2a- scramble shRNA-mCherry. AddGene. https://www.addgene.org/245064/. Deposited 12 August 2025.

