# Supplementary material for: The locus coeruleus maintains core body temperature and protects against hypothermia during dexmedetomidine-induced sedation
Source: Proc Natl Acad Sci U S A. 2025 Oct 7;122(41):e2422878122. doi: 10.1073/pnas.2422878122 (PMC12541345; doi:10.1073/pnas.2422878122)
Supplement: Supplementary file 1 — Appendix 01 (PDF) [file pnas.2422878122.sapp.pdf]

Supporting Information for:

The locus coeruleus maintains core body temperature and protects against hypothermia during dexmedetomidine-induced sedation

Berta Anuncibay Soto, Ying Ma, Mathieu Nollet, Sara Wong, Giulia Miracca, Daniel Rastinejad, Raquel Yustos, Alexei L. Vyssotski, Nicholas P. Franks*, William Wisden*

*Correspondence

n.franks@imperial.ac.uk or w.wisden@imperial.ac.uk

Department of Life Sciences & UK Dementia Research Institute, Imperial College London, Exhibition Road, SW7 2AZ, United Kingdom.

This pdf file includes:

Figures S1 to S6

Materials and Methods

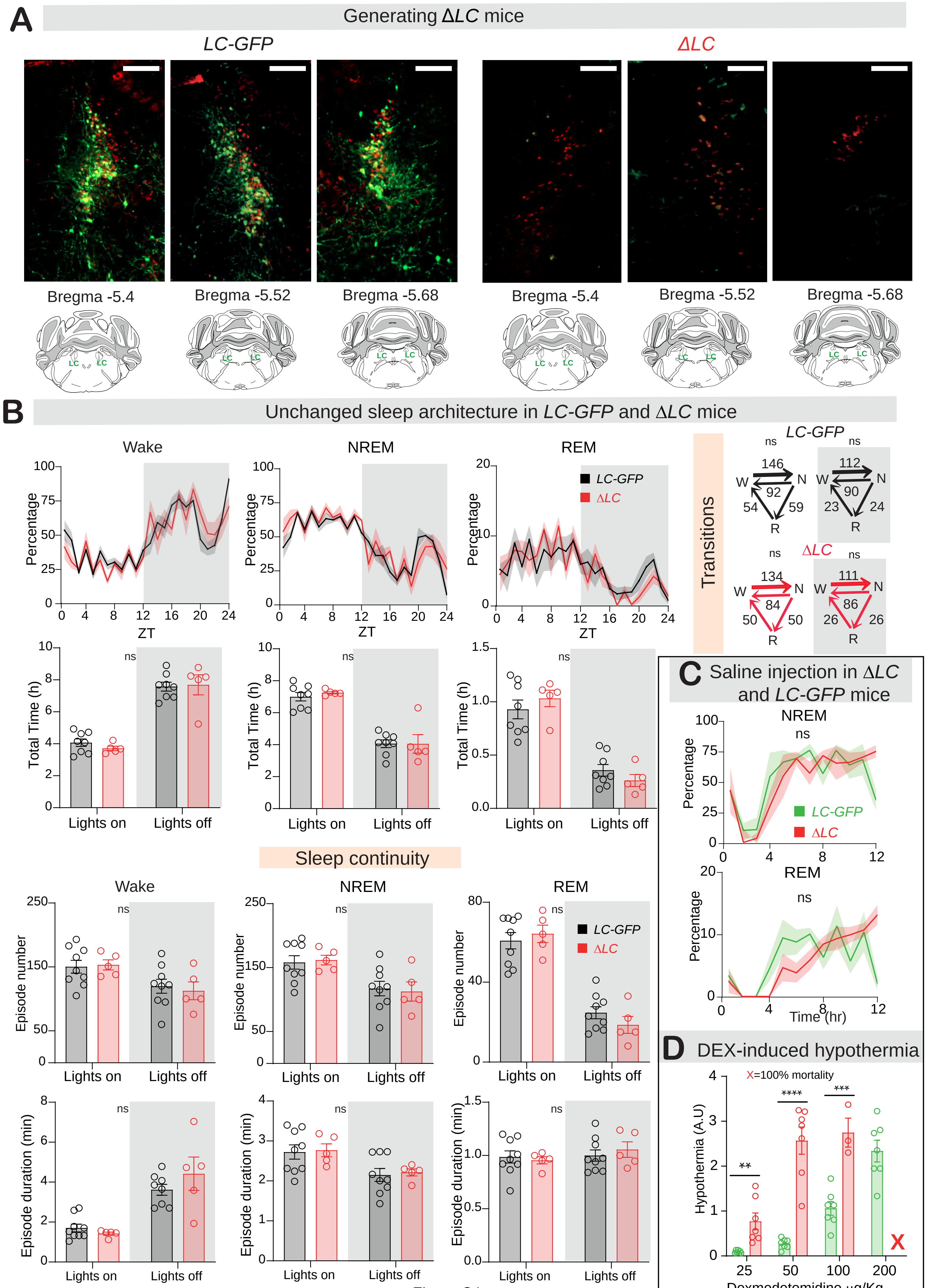
A
Generating ΔLC mice
LC-GFP
ΔLC
Bregma -5.4
Bregma -5.52
Bregma -5.68
LC
B
Unchanged sleep architecture in LC-GFP and ΔLC mice
Wake
NREM
REM
Percentage
ZT
LC-GFP
ΔLC
Transitions
W
N
R
ns
Total Time (h)
Lights on
Lights off
Sleep continuity
Episode number
Episode duration (min)
C
Saline injection in ΔLC and LC-GFP mice
Time (hr)
D
DEX-induced hypothermia
X=100% mortality
Hypothermia (A.U)
Dexmedetomidine µg/Kg

Figure S1

Figure S1. Characterization of LC neuron lesioning and unaltered sleep-wake baseline in *ΔLC* animals. (Supports Fig. 1 and Fig. 2).

*(*A) Representative series of coronal sections showing neuron ablation in the LC of *ΔLC* mice (right-hand panels); ablation is indicated by the absence of GFP- positive staining (since *AAV-DIO-GFP* was co-transduced with *AAV-DIO-caspase* into the LC of *Gal-Cre* mice) and a large reduction in tyrosine hydroxylase-positive neurons in the LC. Normal levels of tyrosine hydroxylase-positive neurons and GFP positive neurons after AVV injection in the LC are shown in *LC-GFP* mice (left-hand panels). Scale bar, 100µm.

(*B*) Sleep architecture was not altered by the ablation of LC neurons. Amounts of WAKE, NREM and REM expressed in percentage (curves), total time (bar graphs) (2-way ANOVA, ns not significant) and sleep transitions in *ΔLC* (N=5) and *LC-GFP* (N=8) mice (2-way ANOVA, ns, not significant). Similarly, sleep consolidation was not altered by the ablation of LC neurons. Episode number and episode duration of WAKE, NREM and REM (2-way ANOVA, ns not significant) in *ΔLC* (N=5) and *LC-GFP* (N=9) mice.

(C) Comparing *ΔLC* and *LC-GFP* mice, saline injections had no effect on inducing sleep.

(D) Bar graph shows the areas (body temperature x time) between the “DEX” and “saline” curves, indicating the extent of hypothermia (unpaired t-test for each dose; ** P<0.01; *** P<0.005; **** P<0.001). Red “X” indicates 100% mortality.

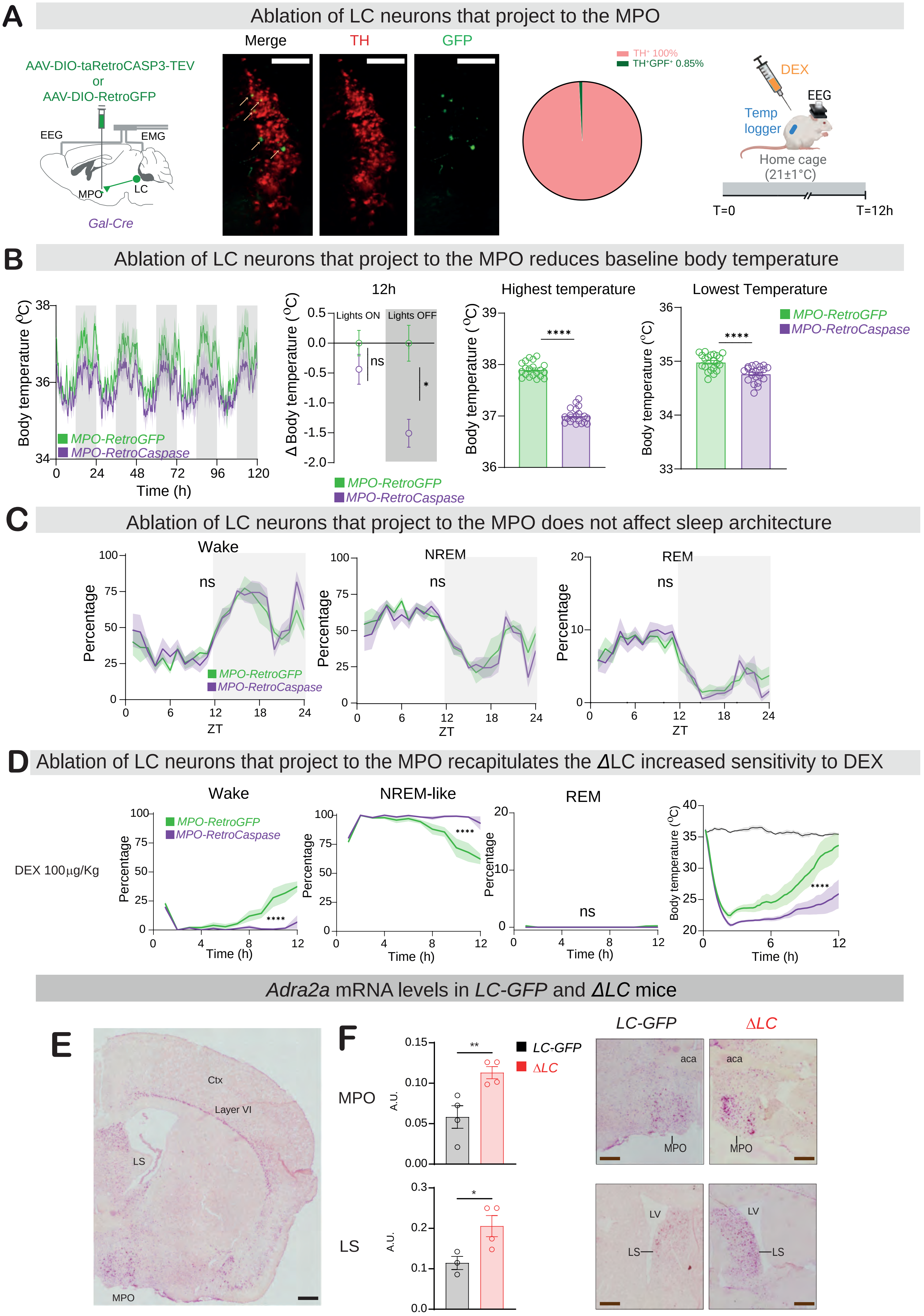
A
Ablation of LC neurons that project to the MPO
AAV-DIO-taRetroCASP3-TEV
or
AAV-DIO-RetroGFP
EEG
EMG
MPO
LC
Gal-Cre
Merge
TH
GFP
TH+ 100%
TH+GPF+ 0.85%
DEX
EEG
Temp
logger
Home cage
(21±1°C)
T=0
T=12h
B
Ablation of LC neurons that project to the MPO reduces baseline body temperature
Body temperature (°C)
Time (h)
MPO-RetroGFP
MPO-RetroCaspase
12h
Lights ON
Lights OFF
Δ Body temperature (°C)
ns
*
Highest temperature
Lowest Temperature
****
C
Ablation of LC neurons that project to the MPO does not affect sleep architecture
Wake
NREM
REM
Percentage
ns
ZT
D
Ablation of LC neurons that project to the MPO recapitulates the ΔLC increased sensitivity to DEX
DEX 100µg/Kg
Wake
NREM-like
REM
Percentage
Time (h)
Body temperature (°C)
****
ns
Adra2a mRNA levels in LC-GFP and ΔLC mice
E
Ctx
Layer VI
LS
MPO
F
MPO
LS
A.U.
**
*
LC-GFP
ΔLC
aca
LV

Figure S2

Figure S2. The LC maintains body temperature by projections to the midline and MPO hypothalamus, and these projections reduce the sensitivity of mice to DEX; and *Adra2* mRNA expression in *LC-GFP* control and *ΔLC* mice. (Supports Figs 2 and 5).

(A) *Left panel:* Schematic illustrating LC *gal*-expressing neurons projecting to the MPO, labelled via *AAV-DIO-RetroGFP* injection into the MPO. Representative coronal section images show GFP-positive neurons colocalized with tyrosine hydroxylase (TH)-positive neurons in the LC. Scale bar: 100 µm.

Middle panel: Quantification of GFP-positive neurons as a percentage of TH-positive neurons in the LC. Right panel schematic of the experimental design. EEG: electroencephalogram

(B) Lesioning LC neurons that project to the MPO reduces body temperature as assayed over multiple days (n=5 per group); middle, average decrease in body temperatures of *MPO-RetroCaspase* mice compared with *MPO-RetroGFP* controls during lights off (n = 5, unpaired t-test, *P < 0.05); and right, bar graphs of the highest and lowest temperatures varying over the 24 hour cycle, (n=5 per group, 20 highest or lowest temperature reached over 5 days) Unpaired two-tailed t -test, ****P < 0.001.

(C) Sleep architecture was not altered by the ablation of LC neurons that project into MPO. Amounts of WAKE, NREM and REM expressed in percentage curves (n=5 per group) 2-way ANOVA, ns, not significant.

(D) Lesioning of LC neurons that project into MPO recapitulates *ΔLC* sensibility to DEX. Amounts of wake, NREM-like sleep and REM sleep over time following DEX injection of 100µg/kg in *MPO-RetroCaspase* (n=5) and *MPO-RetroGFP* (n=5) mice (2-way ANOVA, ****P<0.001). Shading indicates, s.e.m.

(E), *adar2a* mRNA distribution, as detected by RNAscope, in a coronal brain section from an adult control mouse. Ctx, neocortex; LS, lateral septum, MPO, medial preoptic hypothalamus, VI, layer 6 pyramidal layer; scale bars, 1000µm (black), 100µm (brown).

(F) area quantification of *adra2a* mRNA signals in MPO hypothalamus and lateral septum in control and *ΔLC* mice, and RNAscope images showing increased *adra2a* transcript levels in the MPO and LS areas of *ΔLC* mice. Bar graphs, 2 tailed t-test, * $P<0.05$; ** $P< 0.01$ (n= 4 mice per group). A.U. arbitrary units.

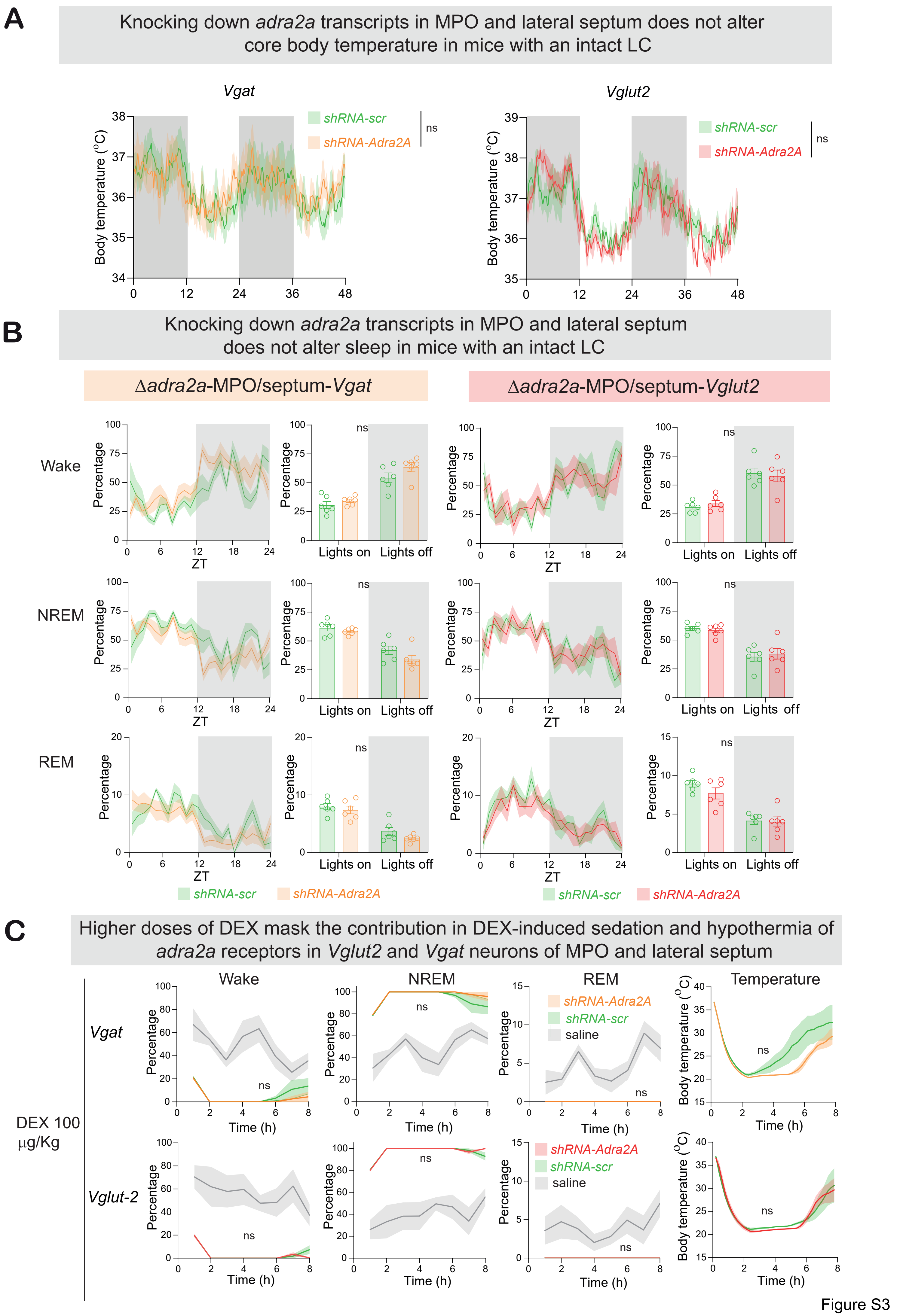
A
Knocking down adra2a transcripts in MPO and lateral septum does not alter core body temperature in mice with an intact LC
Vgat
Vglut2
shRNA-scr
shRNA-Adra2A
ns
Body temperature (°C)
B
Knocking down adra2a transcripts in MPO and lateral septum does not alter sleep in mice with an intact LC
Δadra2a-MPO/septum-Vgat
Δadra2a-MPO/septum-Vglut2
Wake
NREM
REM
Percentage
ZT
Lights on
Lights off
ns
shRNA-scr
shRNA-Adra2A
C
Higher doses of DEX mask the contribution in DEX-induced sedation and hypothermia of adra2a receptors in Vglut2 and Vgat neurons of MPO and lateral septum
Wake
NREM
REM
Temperature
Vgat
Vglut-2
DEX 100 µg/Kg
shRNA-Adra2A
shRNA-scr
saline
Percentage
Body temperature (°C)
Time (h)
ns

Figure S3

Figure S3. Baseline sleep and core body temperature of *Δadra2a-PO/septum-Vglut2* and *Δadra2a-PO/septum-Vgat* mice (Supports Fig 5).

(*A*) Knocking down *adra2a* receptor transcripts in GABA or glutamatergic neurons of the LS and PO did not alter core body temperature of *Δadra2a-PO/septum* (n=6) and control (scr) (n=6) mice (2-way ANOVA, ns, significant).

(*B*) Sleep amounts were not altered by knocking down *adra2a* receptor transcripts in glutamatergic or GABA neurons in the LS and PO brain areas (left-hand panels). *Δadra2a-PO/septum* (n=6) and control (scr) (n=6) mice (2-way ANOVA, *P<0.05, ns, not significant).

(*C*) Sleep amounts or temperature were not altered by knocking down *adra2a* receptor transcripts in glutamatergic or GABA neurons in the LS and PO brain areas when high doses of DEX (100 μg/kg) were administered. *Δadra2a-PO/septum* (n =3) and control (scr) (n =3) mice (2-way ANOVA, ns, not significant).

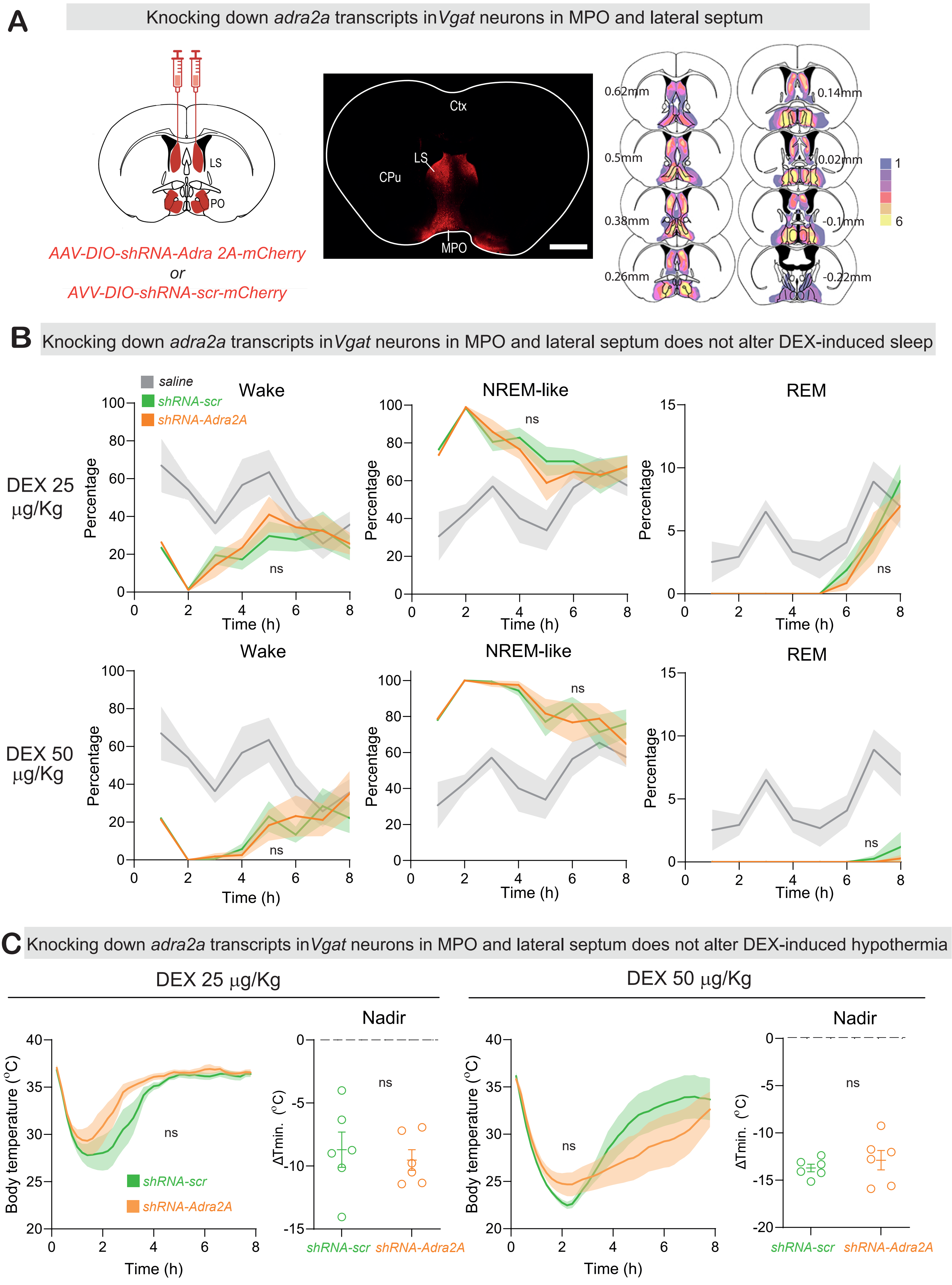

A
Knocking down adra2a transcripts in Vgat neurons in MPO and lateral septum
LS
PO
AAV-DIO-shRNA-Adra 2A-mCherry
or
AVV-DIO-shRNA-scr-mCherry
Ctx
LS
CPu
MPO
0.62mm
0.5mm
0.38mm
0.26mm
0.14mm
0.02mm
-0.1mm
-0.22mm
1
6
B
Knocking down adra2a transcripts in Vgat neurons in MPO and lateral septum does not alter DEX-induced sleep
saline
shRNA-scr
shRNA-Adra2A
DEX 25 µg/Kg
DEX 50 µg/Kg
Wake
NREM-like
REM
Percentage
Time (h)
ns
C
Knocking down adra2a transcripts in Vgat neurons in MPO and lateral septum does not alter DEX-induced hypothermia
DEX 25 µg/Kg
DEX 50 µg/Kg
Nadir
Body temperature (°C)
ΔTmin. (°C)
Time (h)
shRNA-scr
shRNA-Adra2A
ns

Figure S4

Figure S4. DEX does not induce hypothermia or NREM-like sleep via *adra2a* receptors expressed on GABA neurons in the PO hypothalamus/lateral septum (supports Fig. 5).

(*A*) Left-hand panel: Schematic of knockdown of *adra2a* receptor transcripts in the LS and PO of *Vgat-Cre* mice. Middle panel: expression of *AAV-adra2a-shRNA* transgene in lateral septum (LS) and preoptic area (PO), as detected by mCherry immunocytochemistry (coronal section). Color key indicates the number of mice that had transgene expression in the area. Right-hand panel: heatmap documenting the pooled distribution of *AAV-adra2a-shRNA* transgene expression in n=6 mice, as detected by mCherry staining. CPu, Caudate-putamen; Ctx, Cortex. Scale bar, 1000μm

(*B*) DEX-induced sedation does not require *adra2a* receptors in GABA neurons of the PO and LS. Amounts of wake, NREM-like sleep and REM sleep over time following saline or ascending doses of DEX in *Δadra2a-PO/septum-Vgat* (n=5) and controls (scr) (n=5) mice (2-way ANOVA, ns not significant). Shading indicates, s.e.m.

(*C*) DEX-induced hypothermia produced with 25 μg/kg and 50 μg/kg DEX does not require *adra2a* receptors in GABA neurons in the PO and LS. Curves show temperature v. time graphs for 8 hours after DEX injection (2 way-ANOVA, ns, not significant); graphs show the minimum body temperature (nadir) induced by each dose of DEX, which occurred at approx. 2 hours after injection (non-paired t-test, ns, not significant). For each dose, DEX in *Δadra2a-PO/septum-Vgat* (n=6) and controls (scr) (n=6) mice.

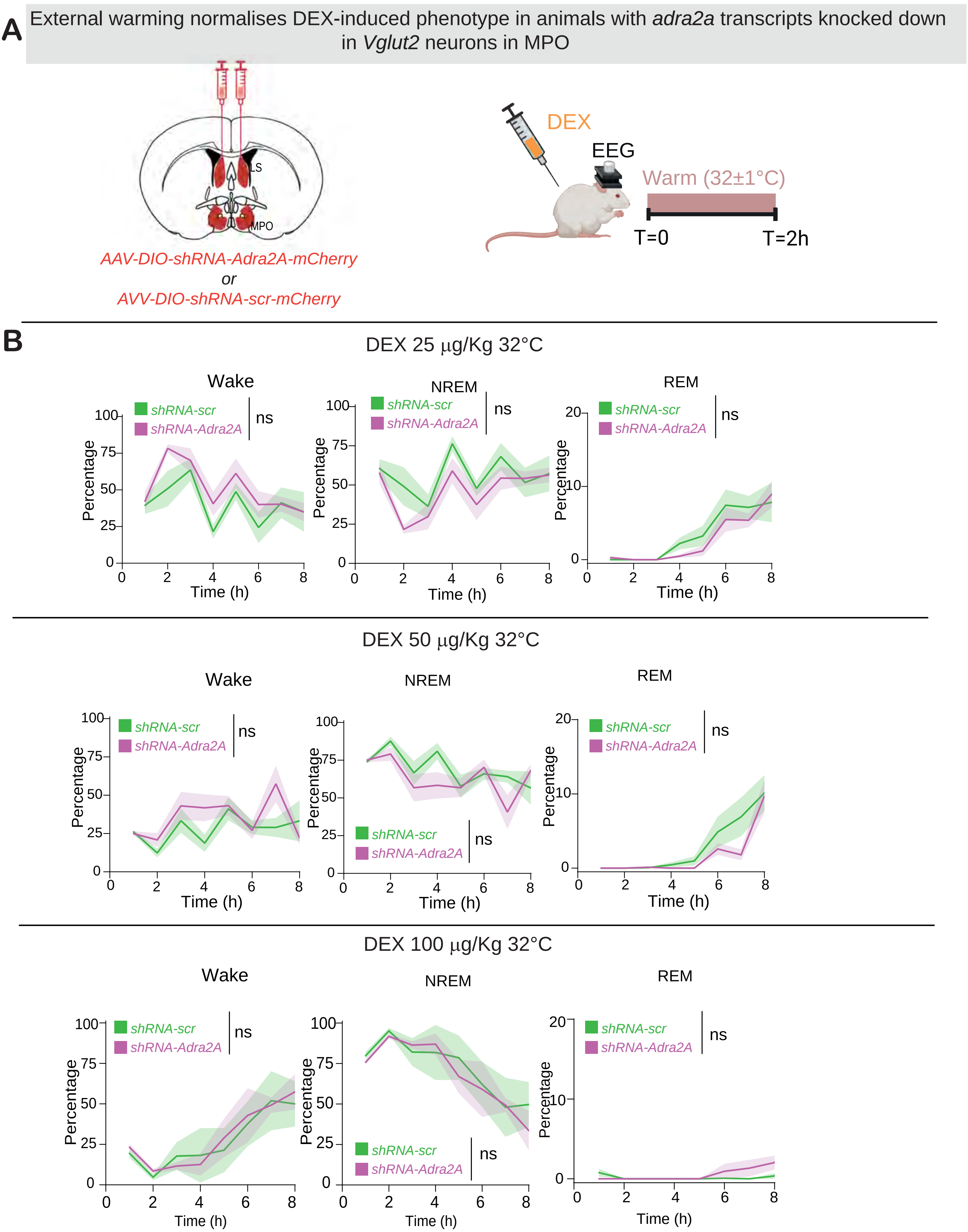
A
External warming normalises DEX-induced phenotype in animals with adra2a transcripts knocked down in Vglut2 neurons in MPO
LS
MPO
AAV-DIO-shRNA-Adra2A-mCherry
or
AVV-DIO-shRNA-scr-mCherry
DEX
EEG
Warm (32±1°C)
T=0
T=2h
B
DEX 25 µg/Kg 32°C
Wake
NREM
REM
shRNA-scr
shRNA-Adra2A
ns
Percentage
Time (h)
DEX 50 µg/Kg 32°C
DEX 100 µg/Kg 32°C

Figure S5

Figure S5. ***Adra2a* receptors on *vglut2*-expressing MPO neurons predominantly regulate DEX-induced hypothermia rather than NREM-like sleep.**

A, Left-hand panel: Schematic of knockdown of *adra2a* receptor transcripts in the MPO area of *Vglut-2-Cre* mice and experimental design. EEG, electroencephalogram. **B**, Amounts of wake, NREM-like sleep and REM sleep over time following ascending doses of DEX injections maintaining the animals in a warm environment for the first 2h, in *Δadra2a-MPO/septum-Vglut-2* (n=5) and controls (scr) (n=5) mice (2-way ANOVA, ns, not significant). Shading indicates, s.e.m.

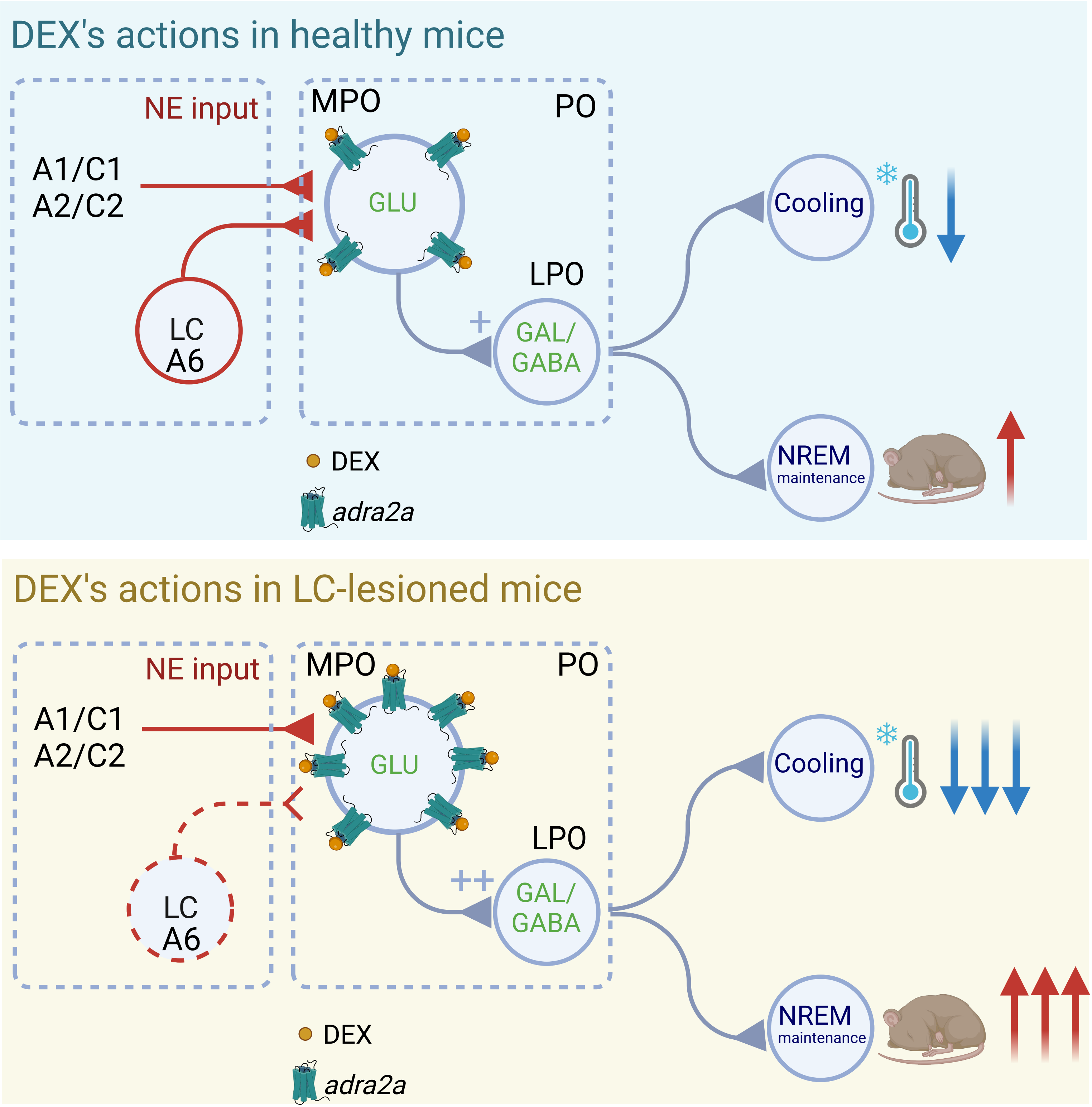
DEX's actions in healthy mice
NE input
A1/C1
A2/C2
LC
A6
MPO
PO
GLU
LPO
+
GAL/
GABA
Cooling
NREM
maintenance
DEX
adra2a
DEX's actions in LC-lesioned mice
NE input
A1/C1
A2/C2
LC
A6
MPO
PO
GLU
LPO
++
GAL/
GABA
Cooling
NREM
maintenance
DEX
adra2a

Figure S6

Figure S6. Circuit hypothesis of how DEX binding to *adra2a* receptors on PO glutamate neurons lowers temperature and contributes to maintaining NREM-like sleep in normal mice and suggestions for how the hypothalamic circuitry becomes more sensitive to DEX following LC lesions.

SUPPLEMENTARY MATERIALS and METHODS

Mice

All experiments were performed in accordance with the United Kingdom Home Office Animal Procedures Act (1986) and were approved by the Animal Welfare and Ethical Review Body at Imperial College. Mice of both sexes were used in the experiments.

Gal-Cre mice (*Tg(Gal-cre)KI87Gsat/Mmucd*) were generated by GENSAT and deposited at the Mutant Mouse Regional Resource Center, stock #031060-UCD, GENSAT Project (NINDS Contracts N01NS02331 and HHSN271200723701C to The Rockefeller University, New York) (1). Gal-Cre mice were bred and used as heterozygotes.

Vglut2-Cre mice (*Vglut2-ires-Cre: Slc17a6*$^{tm2(cre)Lowl/J}$) and *Vgat-Cre* mice (*Vgat-ires-Cre: Slc32a1*$^{tm2(cre)Lowl/J}$) were kindly provided by B. B. Lowell and purchased from the Jackson Laboratory (JAX stock #016963 and #016962, respectively) (2).

All mice were housed with food and water *ad libitum* and maintained under the same conditions (approx. 21 °C, reversed 12/12 h light/dark cycle). Zeitgeber time (ZT) 0 was defined as the time when the light was switched on (4 P.M.) and ZT12 was defined as the start time of lights-off (4 AM). For behavioral and EEG experiments, mice were singly housed, and experiments performed during the dark phase (ZT12–ZT24).

Transgenes and AAVs

We used the following *pAAV* transgene plasmids:

pAAV-DIO-GFP was a gift from John T. Gray (Addgene plasmid 32396).

pAAV-EF1α-DIO-taCASP3-TEV was a gift from Nirao Shah (Addgene plasmid 45580) (3).

pAAV-DIO-hM4Di-mCherry was a gift from Bryan L. Roth (Addgene plasmid 44362) (4).

pAAV-DIO-adra2a-shRNA-mcherry: We used the pPRIME system for shRNA-based knockdown (5). This produces micro-RNA-30 (mir30)-derived shRNAs and co-

expression of a marker protein, such as dsRED or mCherry, from the same transcript (5). To create *pAAV-DIO-adra2a-shRNA-mcherry*, the insert *dsRed-adra2a.shRNA* was generated by PCR from a previous pPRIME-based construct we made, *pAAV-dsRed-adra2a.shRNA* (Addgene plasmid 67880, (6)), introducing an *NheI* site in the forward primer and an *AscI* site in the reverse primer. This PCR product was then double-digested with *NheI* and *AscI* and ligated with a plasmid backbone, *pAAV-hsyn-loxP* (obtained from *pAAV-DIO-hM4Di-mCherry* (4)), after double digestion with *NheI* and *AscI* and gel purification of the 4.8kb vector backbone *pAAV-hsyn-LoxP* fragment. To create *pAAV-DIO-adra2a-shRNA-mcherry*, a *SacI* site was introduced by mutagenesis just before the *DIO-dsRed* reading frame in *pAAV-hsyn-DIO-dsRed-adar2a.shRNA*. This mutated construct was double-digested with *SacI* and *NheI* to remove the *dsRed* fragment and the rest of the plasmid backbone (5.2kb) was gel purified. The *mCherry* coding region was generated by PCR from a plasmid that contained an *mCherry* reading frame, introducing *NheI and SacI* sites in the forward and reverse primers respectively. This *mCherry* PCR product was double-digested with both enzymes and ligated to the previously gel purified backbone, *AAV-hsyn-DIO-SacI-…-NheI-adar2a.shRNA* (5.2kb) fragment. *pAAV-DIO-scramble shRNA-mCherry* was made similarly but starting instead with the plasmid *pAAV-actin prom-dsRed-scramble shRNA* (Addgene plasmid 67879, (6)). The *pAAV-DIO-adra2a-shRNA-mcherry* and *pAAV-DIO-adra2a-scramble shRNA-mCherry* plasmids and their sequences have been deposited at Addgene (Addgene IDs 245063 and 245064 respectively).

All AAVs (serotype 1/2) were produced in-house. The adenovirus helper plasmid *pFΔ6*, the AAV helper plasmids *pH21* (AAV1) and *pRVI* (AAV2), and *pAAV* transgene plasmids listed above were co-transfected into HEK293 cells and the resulting AAVs collected on heparin columns, as described previously (7). To prepare the *AAV-DIO-retro-caspase* serotype, HEK293 cells were co-transfected with the rAAV2 packaging plasmid, a gift from Alla Karpova and David Schaffer (Addgene plasmid #81070) (8),

the helper plasmid *pFΔ6*, together with *pAAV-EF1α-DIO-taCASP3-TEV* or *pAAV-DIO-GFP*, and the resulting AAVs were collected on a heparin column.

Surgeries

Mice were anaesthetized by inhalation with 4% isoflurane and maintained with 2% isoflurane in oxygen on a stereotaxic frame (Angle Two, Leica Microsystems, Milton Keynes, Buckinghamshire, UK). AAVs were injected bilaterally through stainless steel 33-gauge/15mm/PST3 internal cannulas (Hamilton) attached to a 10 µl Hamilton syringe, at a rate of 0.1 µl per min.

The injection volumes and coordinates were:

LC: median-lateral ML (± 0.86mm), anterior-posterior AP (-5.40mm), dorso-ventral DV (-3.80mm) 150 nl + 150 nl.

LS: median-lateral ML (± 0.45mm), anterior-posterior AP (+0.50mm), dorso-ventral (DV -3.00mm) 200 nl + 200 nl.

MPO: median-lateral ML (± 0.75mm), anterior-posterior AP (+0.20mm), dorso-ventral (DV -5.70mm) 200 nl + 200 nl.

After virus injection, two EEG screw electrodes were chronically implanted and placed relative to bregma at AP: −1.50 mm, ML: −2.00 mm; AP: +1.50 mm, ML: −2.00 mm on the skull of the mice, moreover in the extensor muscle of the neck two EMG wire electrodes (AS634, Coorner Wire) were inserted.

EEG/EMG recordings and analysis

Neurologger 2A devices, set at a sampling rate of 200 Hz, were used to record EEG and EMG signals (9). The data were visualized with Spike2 software (Cambridge Electronic Design). Offline EEG signals were high-pass filtered at 0.5 Hz (−3 dB), similarly EMG signals were bandpass filtered at 5–45 Hz (−3 dB). EMG integral, delta power (0.5–4.5 Hz), theta power (5–10 Hz) and theta/delta (T:D) ratios were calculated to determine the vigilance states (wake, NREM and REM sleep). Results were manually corrected (after automated sleep scoring was performed by Spike2).

Sleep Scoring

Initial sleep scoring was automated, using the Spike2 version 11 algorithm specifically configured for murine sleep analysis. Electroencephalographic (EEG) signals were segmented into 5-second epochs. Thresholds for delta power, theta/delta ratio, and electromyographic (EMG) activity were manually adjusted for each individual recording.

Vigilance states were classified based on the following criteria:

NREM and “NREM-like Sleep”: Defined by high-amplitude EEG activity (predominantly delta waves) and absent or low EMG activity. We found in another study that as chemogentically-evoked hypothermia in mice progressed from 37°C to an average depth of 29°C, there was an overall 40% reduction in EEG amplitude and a similar percentage reduction in peak delta (1–4 Hz) frequency(10). Even in this lower power EEG state, however, the mice could still clearly be scored as being in a NREM-like state (high ratio of delta to theta power in the EEG paired with little activity in the EMG; see Figures 3F and 3G), as also found by others(11). This is how we scored the NREM-like states that occurred with hypothermia following DEX administration.

REM Sleep: Defined by theta-frequency EEG activity and absence of EMG activity.

WAKE: Defined by high-frequency EEG activity accompanied by high EMG activity.

Epochs that did not meet both criteria for any specific state were designated as "Doubt" by the algorithm. After automated scoring, each dataset was reviewed manually, with particular attention given to epochs flagged as “Doubt”.

Although certain epochs were excluded from analysis in some sleep recordings due to signal artifacts, no unscored epochs attributable to burst suppression or isoelectric periods were observed.

Temperature Recordings

Core body temperature was measured using temperature loggers (DST nano, Star-Oddi) pre-programmed offline to sample temperature data every 3 min for all the experiments. Loggers were intra-abdominally implanted as described previously (10). Temperature measurements were retrieved and analyzed offline.

Dexmedetomidine (DEX) injections

Mice with implanted temperature loggers were fitted with Neurologger 2A devices the night before the experiment. At ZT 18, DEX (Tocris Bioscience), dissolved in saline to make a final concentration of 25, 50, 100 or 200 μg/kg, was delivered *i.p.* Immediately after injection, and mice were then placed into a behavioral area or a warm environment (32°C) for 2h and then back to their home cage, or directly back to their home cage. EEG/EMG data and core body temperature were simultaneously recorded. A baseline recording from the same mouse, injected with saline, of its baseline sleep-wake cycle and core body temperature in the same circadian time were used for parallel comparison with the DEX injection experiments.

Chemogenetics

At ZT 18 (*i.p.* injection t=0) in the middle of the active period (lights-off) when mice were most active and least likely to sleep, mice were injected with DEX100μg/kg and the vigilance states were recorded. One hour before DEX injection, CNO (4936, Tocris) dissolved in saline at 1 mg/kg, or saline alone, was injected *i.p.* Mice receiving saline or CNO injections at the same circadian time were randomized. To avoid additional stress for the mice, Neurologger 2A devices were placed onto the animals the day before.

Histology and Immunostaining

Mice were transcardially perfused with 4% paraformaldehyde in phosphate-buffered saline (Sigma-Aldrich). Coronal 40µm sections were cut on a freezing microtome (Thermo Scientific HM 450 Sliding Microtome).

For antigen retrieval sections in free floating were transferred to a sodium citrate buffer (10mM sodium citrate (Sigma-Aldrich) 0.05% TWEEN® (Sigma-Aldrich), pH 6.0) and incubated at 85° C for 30 minutes. Sections were washed 3 times in PBS for 10 minutes and then, blocked in 20% normal goat serum (NGS) (Vector), PBS, 0.2% Triton™ X-100 (Sigma-Aldrich) for 1 hour at room temperature (RT). Sections were incubated with primary antibody overnight at 4°C in 2% NGS, PBS, 0.2% Triton™ X-100. Next day (all at RT), sections were washed in PBS 3 times for 10 minutes, incubated with secondary antibody for 1.5 hours in 2% NGS, PBS, 0.2% Triton™ X-100. After 3 washes in PBS, sections were mounted onto Superfrost® Plus slides (ThermoFisher) with ProLong™ Gold antifade reagent (ThermoFisher). Primary antibodies were chicken anti-GFP (Abcam, ab13970, 1:1000), rat monoclonal anti-mCherry (Sigma-Aldrich, M11217, 1:1000), rabbit polyclonal anti-mCherry (Abcam, ab183628, 1:1000), mouse monoclonal anti-tyrosine hydroxylase (TH) (Sigma-Aldrich, T2928, 1:1000). Secondary antibodies were Alexa Fluor 594 goat anti-rat (Invitrogen, A11007, 1:500), Alexa Fluor 488 goat anti-chicken (Invitrogen, A11039, 1:500), Alexa Fluor 488 goat anti-mouse (Invitrogen, A11029, 1:500), Alexa Fluor 594 goat anti-rabbit (Invitrogen, A11037, 1:500). Samples were imaged using an Inverted Widefield Microscope (Zeiss Axio Observer), and with a Leica SP8 Inverted confocal microscope. Channels were imaged sequentially and further analyzed with Fiji version 2.9.0. Figures were assembled using Adobe Illustrator version 27.5.

The number of tyrosine hydroxylase-positive neurons was quantified in 20µm equidistant sections containing the extent of the LC (from -5.40 Bregma to -5.70 Bregma). For quantification, 20x images containing both LCs (*i.e.* on each side of the brain) were acquired using Inverted Widefield Microscope (Zeiss Axio Observer). The total number of visible neurons was counted in four equidistant sections belonging to

four animals per group. Each hemisphere was quantified independently and later the number of neurons averaged per section. The four sections were then averaged per animal.

RNAscope

Tissue collection, pretreatment and *in situ* hybridization was performed according to the RNAscope protocol for frozen tissue (Advanced Cell Diagnostics (ACD)). Probes were provided by ACD: *Adra2a*: Mm Adra2a Cat No. 425341 and *Galanin*: Mm Galanin Cat No. 400961. *Adra2a* transcript signals were quantified in 10μm equidistant sections containing the LS and PO areas (from +0.5 Bregma to -0.10 Bregma). For quantification, 20x images containing both LS and PO were acquired using an Inverted Widefield Microscope (Zeiss Axio Observer). In each image, at least 6 squares were drawn manually in the LS and PO and the areas covered by positive *adra2a* staining were measured. Six to eight sections containing both LS and PO were quantified and later averaged per animal. Four animals per group were analyzed.

Experimental Design and Statistical analysis

No statistical methods were used to pre-determine sample sizes, but our sample sizes are similar to those reported in our previous publications (6, 12). Experimenters were not blinded during treatment administration, but data analysis was performed blind. Mice were excluded from the analysis if the histology did not confirm AAV transgene expression. Prism10 was used for statistical analysis. In the figures data represented are mean ±SEM, NS indicates $P \geq 0.05$, $*P < 0.05$, $**P < 0.01$, $***P < 0.001$, $****P < 0.0001$. Distributions were assumed to be normal. Two-way ANOVA with Sidak correction and the mixed-effects model or non-parametric two-tailed Wilcoxon matched-paired signed-rank test were used depending on the parameters analyzed, and the statistical test performed in each of the graphs is specified in the figure legends.

1. E. F. Schmidt, L. Kus, S. Gong, N. Heintz, BAC transgenic mice and the GENSAT database of engineered mouse strains. *Cold Spring Harb Protoc* **2013** (2013).
2. L. Vong *et al.*, Leptin action on GABAergic neurons prevents obesity and reduces inhibitory tone to POMC neurons. *Neuron* **71**, 142-154 (2011).
3. C. F. Yang *et al.*, Sexually dimorphic neurons in the ventromedial hypothalamus govern mating in both sexes and aggression in males. *Cell* **153**, 896-909 (2013).
4. M. J. Krashes *et al.*, Rapid, reversible activation of AgRP neurons drives feeding behavior in mice. *J Clin Invest* **121**, 1424-1428 (2011).
5. F. Stegmeier, G. Hu, R. J. Rickles, G. J. Hannon, S. J. Elledge, A lentiviral microRNA-based system for single-copy polymerase II-regulated RNA interference in mammalian cells. *Proc Natl Acad Sci U S A* **102**, 13212-13217 (2005).
6. Z. Zhang *et al.*, Neuronal ensembles sufficient for recovery sleep and the sedative actions of alpha2 adrenergic agonists. *Nat Neurosci* **18**, 553-561 (2015).
7. M. Klugmann *et al.*, AAV-mediated hippocampal expression of short and long Homer 1 proteins differentially affect cognition and seizure activity in adult rats. *Mol Cell Neurosci* **28**, 347-360 (2005).
8. D. G. Tervo *et al.*, A Designer AAV Variant Permits Efficient Retrograde Access to Projection Neurons. *Neuron* **92**, 372-382 (2016).
9. V. N. Anisimov *et al.*, Reconstruction of vocal interactions in a group of small songbirds. *Nat Methods* **11**, 1135-1137 (2014).
10. E. C. Harding *et al.*, A Neuronal Hub Binding Sleep Initiation and Body Cooling in Response to a Warm External Stimulus. *Curr Biol* **28**, 2263-2273 e2264 (2018).
11. T. Deboer, I. Tobler, Temperature dependence of EEG frequencies during natural hypothermia. *Brain Res* **670**, 153-156 (1995).
12. Y. Ma *et al.*, Galanin Neurons Unite Sleep Homeostasis and alpha2-Adrenergic Sedation. *Curr Biol* **29**, 3315-3322 e3313 (2019).
